# *In Situ* Characterization of Bak Clusters Responsible for Cell Death Using Single Molecule Localization Microscopy

**DOI:** 10.1038/srep27505

**Published:** 2016-06-13

**Authors:** Yusuke Nasu, Alexander Benke, Satoko Arakawa, Go J. Yoshida, Genki Kawamura, Suliana Manley, Shigeomi Shimizu, Takeaki Ozawa

**Affiliations:** 1 Department of Chemistry, School of Science, The University of Tokyo, 7-3-1 Hongo, Bunkyo-ku, Tokyo 113-0033, Japan; 2 Laboratory of Experimental Biophysics, École Polytechnique Fédérale de Lausanne, CH-1015 Lausanne, Switzerland; 3 Department of Pathological Cell Biology, Medical Research Institute, Tokyo Medical and Dental University, 1-5-45 Yushima, Bunkyo-ku, Tokyo 103-5802, Japan

## Abstract

Apoptosis plays a pivotal role in development and tissue homeostasis in multicellular organisms. Clustering of Bak proteins on the mitochondrial outer membrane is responsible for the induction of apoptosis by evoking a release of pro-apoptotic proteins from mitochondria into cytosol. However, how the protein cluster permeabilizes the mitochondrial membrane remains unclear because elucidation of the cluster characteristics such as size and protein density has been hampered by the diffraction-limited resolution of light microscopy. Here, we describe an approach to quantitatively characterize Bak clusters *in situ* based on single molecule localization. We showed that Bak proteins form densely packed clusters at the nanoscale on mitochondria during apoptosis. Quantitative analysis based on the localization of each Bak protein revealed that the density of Bak protein is uniform among clusters although the cluster size is highly heterogeneous. Our approach provides unprecedented information on the size and protein density of Bak clusters possibly critical for the permeabilization and is applicable for the analysis of different cluster formations.

Intrinsic mechanisms of cell death exist in multicellular organisms. One such mechanism, apoptosis, plays a crucial role in early development, homeostasis, and immunity in organisms^1^. Mitochondria often serve as a central organelle in the induction of apoptosis^2^. In response to external stimuli such as cytotoxic chemicals, starvation, and ultraviolet (UV) light, Bak and Bax proteins oligomerize to form clusters on the mitochondrial outer membrane. These clusters are responsible for the release of pro-apoptotic proteins such as cytochrome c and Smac/DIABLO from mitochondria into the cytosol. The release facilitates activation of caspases, leading to the degradation of proteins and DNA, and finally to cell death. Accordingly, the formation of Bak/Bax clusters on mitochondria is essential for the induction of apoptosis. Multiple studies have revealed the molecular mechanisms involved in the oligomerization of each Bak/Bax monomer^3^,^4^,^5^. However, how these clusters permeabilize the membrane to release the pro-apoptotic proteins remains elusive^2^. It is a subject of considerable debate in this question whether the cluster itself serves as a channel-like pore on the mitochondrial outer membrane (proteinaceous pore model) or the cluster disrupts the membrane to form a lipidic pore (lipidic pore model). Investigation of cluster characteristics in the cellular milieu is indispensable for addressing this question, but *in situ* characterization of the cluster has been impeded by the diffraction-limited resolution of light microscopy^6^. For alternative approaches such as electron microscopy, the labeling efficiency is insufficient to observe a significant fraction of Bak/Bax molecules in a cluster^7^. Such low labeling density hampers quantitative characterization of the cluster. Consequently, the challenge lies in devising a methodology that enables to characterize the Bak/Bax cluster *in situ* in a quantitative manner with sufficient spatial resolution.

Techniques of super-resolution fluorescence imaging enable the acquisition of images with resolution beyond the diffraction limit^8^,^9^,^10^. Of these methods, photoactivated localization microscopy (PALM) uses photoactivatable or photoswitchable probes to specifically visualize individual target molecules *in situ*. PALM provides information on localization of the molecule in a pointillistic fashion, allowing the quantitative analysis of closely spaced structures such as protein clusters. Because of these important features, various studies based on PALM have elucidated nanoscopic protein organizations such as bacterial receptor clustering^11^, virus protein assembly^12^,^13^, and RNA polymerase clustering^14^,^15^.

We herein demonstrate a PALM-based approach for quantitative characterization of Bak clusters *in situ*. Using a functional, genetically encoded probe to specifically detect physiologically relevant Bak clusters in the absence of an endogenous background, we visualized clusters on mitochondria with nanoscale resolution and analyzed their sizes on a single-molecule level. The results provide important characteristics of the Bak cluster largely responsible for apoptosis.

## Results

### Generation of a cell line expressing mEos3-Bak

**Figure 1:**
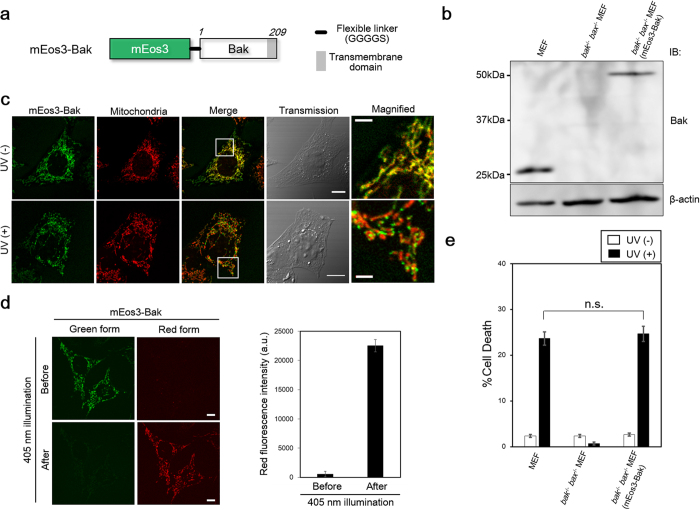
Generation of a cell line expressing mEos3-Bak. (**a**) Schematic construct of mEos3-Bak. mEos3 is fused to Bak at the N-terminus with a flexible linker (Gly, Gly, Gly, Gly and Ser). Numbers in italics show the residue of amino acids in Bak. Transmembrane region for mitochondrial localization is shown as a gray box (189–209). (**b**) Western blot analysis for investigating the expression of mEos3-Bak in the cell line. The bands indicate the expression of mEos3-labeled Bak (49.6 kDa), endogenous Bak (23.3 kDa) and β-actin, respectively. (**c**) Confocal imaging of the cells in the presence or absence of UV irradiation. Mitochondria were visualized by MitoTracker CMXRos dye. Magnified images in white boxes (Merge) are shown (right panels). Scale bars: 10 μm (transmission) or 2 μm (magnified). (**d**) Violet-light-dependent photo-conversion of mEos3-Bak. UV (254 nm) light was irradiated and subsequently fluorescence intensity in red form of mEos3 before and after violet light illumination was measured (right). Scale bars: 10 μm. Data are represented as mean ± s.e.m. (n = 3 cells). (**e**) Trypan blue exclusion assays for examining cell viability upon UV stimulation. Statistical analysis was performed with two-tailed unpaired Student’s *t*-test. In the analysis, *P* > 0.05 is indicated as not significant (n.s.). Data are represented as mean ± s.e.m. (n = 3).

To observe Bak clusters using PALM, Bak was labeled with the photoswitchable fluorescent protein, mEos3 (Fig. 1a). The monomeric property of mEos3 is crucially important to abrogate the aggregation of target proteins^16^. The mEos3 was connected with the N-terminus of Bak because the C-terminal region (189–209 residue, Supplementary Fig. 1) is necessary for insertion into the mitochondrial outer membrane. The replacement of endogenous Bak by mEos3-Bak is prerequisite to addressing the characterization of Bak clusters. Thus, the *bak*^−/−^ *bax*^−/−^ mouse embryonic fibroblast (MEF), in which endogenous *bak* and *bax* genes were knocked out, was retrovirally infected with the *mEos3*-*Bak* gene. Subsequently, a single clone was selected by limiting dilution. We confirmed that endogenous Bak was replaced by mEos3-Bak in the cells (Fig. 1b). The subcellular localization of mEos3-Bak was merged with the localization of mitochondria in the absence of apoptotic stimulation, revealing that labeling with mEos3 did not interfere with the mitochondrial localization (Fig. 1c, upper panel). In contrast, mEos3-Bak upon UV irradiation showed a punctate localization on mitochondria, indicative of the formation of Bak clusters (Fig. 1c, lower panel). These Bak clusters colocalized with Bax clusters in apoptotic cells, whereas the Bak did not colocalize with Drp1 under either healthy or apoptotic conditions (Supplementary Fig. 2). Unlike the UV light (254 nm), violet light (405 nm) specifically converted mEos3 from green to red (Fig. 1d), ensuring that the probe is applicable to PALM for imaging Bak clusters during UV-induced apoptosis. In addition, mEos3-Bak rescued the loss of apoptotic function in *bak*^−/−^ *bax*^−/−^ MEF (Fig. 1e). This result demonstrated that mEos3-Bak retained the ability to induce apoptosis.

**Figure 2:**
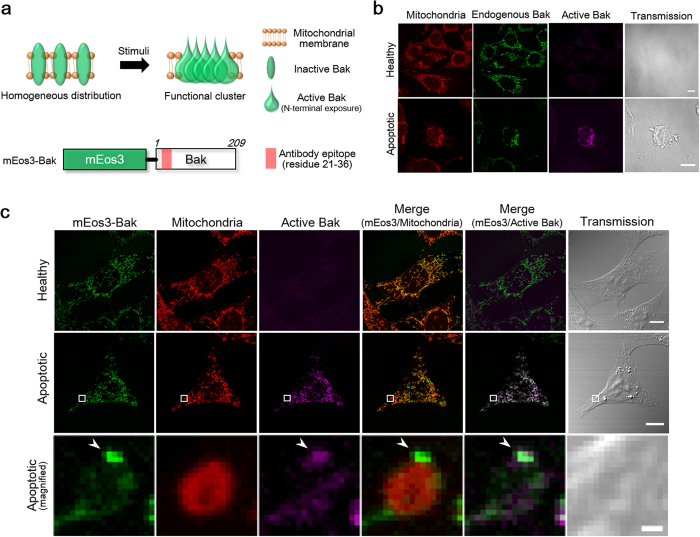
Validation of the intramolecular conformational change in mEos3-Bak. (**a**) Formation of a functional Bak cluster via an intramolecular conformational change. A red box in the schematic structure of mEos3-Bak indicates the epitope recognized by a Bak antibody used in the immunostaining. The epitope in N-terminal region is cryptic in inactive Bak, but exposed upon apoptotic signals to be active Bak. (**b**) Immunostaining of endogenous Bak and active Bak in the wild-type MEF. Scale bars: 10 μm. (**c**) Immunostaining of active Bak in the engineered cell line expressing mEos3-Bak. Magnified images in white boxes (middle) are shown at the bottom. Arrowheads indicate a functional Bak cluster. Scale bars: 10 μm (top and middle) or 0.5 μm (bottom).

Next, we investigated whether the Bak protein fusion to mEos3 in the clusters undergoes a structural change to an active form responsible for the induction of apoptosis. Upon exposure to an apoptotic stimulus, N-terminal residues hidden in the Bak structure are exposed, followed by the formation of a Bak cluster (Fig. 2a)^2^. This structural transition was analyzed by fluorescence immunostaining with an antibody specific for the N-terminus of Bak (Fig. 2b). A fluorescence signal corresponding to active Bak was clearly detected in UV-stimulated cells, whereas no active Bak was found in the unstimulated cells (Fig. 2c). Localization of the active Bak followed the same distribution as that of mEos3-Bak, indicating that mEos3-Bak in the clusters was an active form. Consequently, we concluded that the MEF cells stably expressing mEos3-Bak can be used for visualizing the physiologically relevant Bak clusters *in situ*.

### Super-resolution imaging of Bak clusters

**Figure 3:**
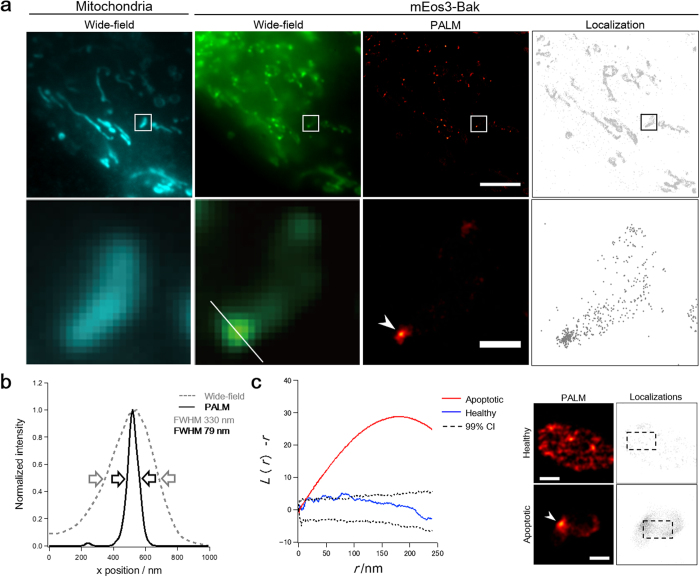
Super-resolution imaging of mEos3-Bak clusters. (**a**) Images of mitochondria (Wide-field) and mEos3-Bak (Wide-field, PALM and Localization). Mitochondria were visualized by MitoTracker Deep Red dye. Magnified images in the boxed regions are shown in lower panels. An arrowhead indicates a Bak cluster. Scale bars: 3 μm (top) or 500 nm (bottom). (**b**) Line-intensity profile of a white line in (**a**). The cluster size was estimated by calculating full width half maximum (FWHM) of the profile. (**c**) Ripley’s K analysis for measuring the degree of the spatial distribution of Bak molecules. *L*(*r*)-*r* was plotted as a function of a radial scale, *r*. The analyses were performed in the boxed regions (480 nm × 800 nm) of a heathy cell and an apoptotic cell. An arrowhead indicates a Bak cluster. CI, confidence intervals. Scale bars: 500 nm.

To observe Bak clusters, which contribute to the induction of apoptosis, we identified the stage at which pro-apoptotic proteins were released from mitochondria into the cytosol (Supplementary Fig. 3). In this stage, Bak clusters are formed on mitochondria without any alteration of the cell morphology. The established cell line, which stably expressed mEos3-Bak, was irradiated with UV and was subsequently fixed with paraformaldehyde at the specified stage. Bak clusters were then observed by PALM, with a mean localization precision of 19.6 nm (Fig. 3a and Supplementary Fig. 4a,d). For a characteristic cluster, the full-width half maximum (FWHM) of the line intensity profile was 330 nm in the wide-field image, whereas that in the PALM image was 79 nm (Fig. 3b). To investigate whether the fixation of the cells influences on the distribution of Bak proteins, we implemented live-cell super-resolution imaging using the stimulated emission depletion (STED) microscopy (Supplementary Fig. 5). The Bak oligomer showed a cluster structure in living apoptotic cells, which was consistent with the result in fixed apoptotic cells. The results indicate that the structure of Bak oligomer was little affected by fixation of the cells. PALM, in combination with electron microscopy, revealed that the ultrastructure of mitochondrial membranes was altered in the cells upon formation of Bak clusters (Supplementary Fig. 6), suggesting that the Bak clustering was correlated with the perturbation of the integrity of the mitochondrial membrane^17^.

PALM provided the locations of individual mEos3-Bak molecules, from which a super-resolution image was reconstructed (Fig. 3a). In the distribution of mEos3-Bak in apoptotic cells, we found that many mEos3-Bak molecules contributed to the formation of clusters, whereas the other molecules appeared dispersed. This biased distribution of mEos3-Bak suggests that the cell undergoes apoptosis before all mEos3-Bak molecules incorporate into clusters. To analyze the spatial distribution of mEos3-Bak molecules, we implemented Ripley’s K analysis to measure the degree of the clustering. The distribution of mEos3-Bak in a healthy cell was random, whereas that of mEos3-Bak in an apoptotic cell showed heterogeneity on the mitochondria (Fig. 3c). Results of the analysis also suggested that mEos3-Bak molecules formed clusters by markedly changing the distribution during apoptosis.

### Characterization of Bak clusters

**Figure 4:**
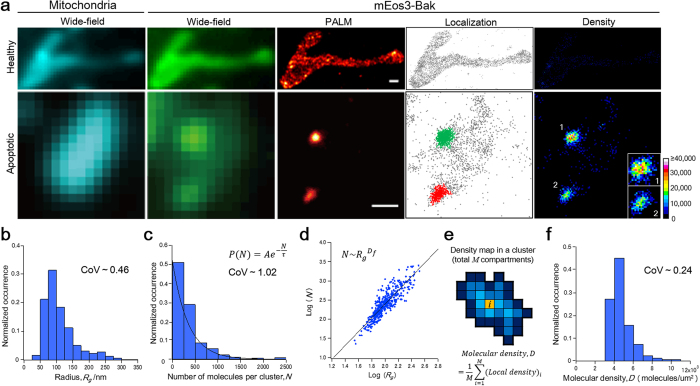
Characterization of mEos3-Bak clusters. (**a**) Images of mitochondria (Wide-field) and mEos3-Bak (Wide-field, PALM, Localization and Density) in a healthy cell and an apoptotic cell expressing mEos3-Bak. Localizations identified as a cluster are colored (mEos3-Bak, Localization). The color bar indicates the molecular density (molecules/μm^2^) in the density maps. Magnified images of the clusters are shown in the inset. Note that magnification of images is not the same between healthy and apoptotic cell, whereas the pixel size of each image is the same between them. Scale bars: 500 nm. (**b**) Normalized histogram of the radii, *R*_*g*_, of mEos3-Bak clusters. (**c**) Normalized histogram of the number of mEos3-Bak molecules per cluster, *N*. The distribution was fitted by single exponential decay. (**d**) Fractal analysis. In each cluster, *N* was plotted as a function of *R*_*g*_ in logarithmic scales. Linear regression of the plot yields the slope which corresponds to the fractal dimension, *D*_*f*_. (**e**) Schematic illustration of computation of the molecular density, *D*, in each cluster. Each local density in an area with 20 nm × 20 nm is colored according to the color bar in (**a**). (**f**) Normalized histogram of *D*. CoV represents the coefficient of variation obtained for the respective data. N = 433 clusters from four cells.

To analyze the Bak clusters quantitatively and in an unbiased manner using the list of mEos3-Bak localization coordinates, we automated the identification of which mEos3-Bak molecules constitute a cluster. For this purpose, we used a cluster algorithm based on the local density of proteins (DBSCAN, Supplementary Fig. 7a)^18^. Clusters identified by the algorithm were consistent with those identified by punctate fluorescence in wide-field and PALM images (Fig. 4a).

Next, we analyzed the cluster radius and the number of molecules per cluster. The analysis revealed that the radius (*R*_*g*_) was broadly distributed between 35 nm and 330 nm, resulting in the mean radius of 111 ± 50 nm (s.d.) (Fig. 4b). Notably, 84% of the analyzed clusters were smaller than the diffraction limit. To rigorously quantify the number of molecules, we imaged exhaustively by collecting data until no fluorescence signal was detected (Supplementary Fig. 8a). Furthermore, we plotted the cumulative number of mEos3-Bak molecules in each cluster, confirming that the imaging of the detectable mEos3 in each cluster was saturated (Supplementary Fig. 8c). The mean number of blinking events per mEos3 molecule (*n*_*blink*_), which might engender overcounting errors, was 0.42 (Supplementary Fig. 9). By normalizing to correct the error caused by blinking, we ascertained that the number of molecules per cluster (*N*) exhibited a wide distribution of 23–2,505 molecules (Fig. 4c). The distribution fitted by a single exponential decay given by *e*^−*N*/*τ*^ yielded *τ* of 344 molecules, where 63% of the detected clusters included fewer than *τ* molecules. We also observed a wide distribution of *R*_*g*_ and *N* in each cell (Supplementary Fig. 10), confirming that the Bak clusters in the cells were imaged at the same stage during apoptosis. To investigate the scaling relation between *R*_*g*_ and *N*, we used fractal analysis (Fig. 4d)^19^,^20^. In this analysis, the scaling relation is characterized by the fractal dimension (*D*_*f*_), which corresponds to the slope of linear regression in a radius – molecular number plot. The analysis of *R*_*g*_ and *N* in each Bak cluster yielded *D*_*f*_ of 1.80 ± 0.09 (95% confidence interval). The value *D*_*f*_ is close to 2, indicating that the cluster area increases nearly linearly with the number of molecules, since clusters are constrained to the two-dimensional membrane. Accordingly, the result suggests that a density of mEos3-Bak molecules within mEos3-Bak clusters was nearly homogeneous, although the cluster size was broadly distributed.

To examine whether the densities of mEos3-Bak molecules within clusters are similar among individual clusters, we measured the molecular density in each Bak cluster independently of *R*_*g*_ and *N*. The molecular density (*D*) in each cluster was calculated by averaging the local densities of mEos3-Bak molecules (Fig. 4e). The value of *D* exhibited a distribution of 3,233–10,576 molecules/μm^2^, resulting in the mean of 4.75 × 10^3^ molecules/μm^2^ (Fig. 4f). Because *D* is 1.55 × 10^3^ molecules/μm^2^ in a healthy cell (Supplementary Fig. 4d), the analysis indicated that the molecular density in a cluster is on average three times higher than that in a healthy cell. The coefficient of variation in *D* (0.24) was smaller than that in *R*_*g*_ (0.46) and *N* (1.02), demonstrating that the distribution of *D* exhibited a narrower range than that of *R*_*g*_ and *N*. The narrow distribution of *D* indicates a similar density between individual clusters, which is consistent with the scaling relation found using the fractal analysis for these two-dimensional objects.

### Characterization of Bak mutant clusters

**Figure 5:**
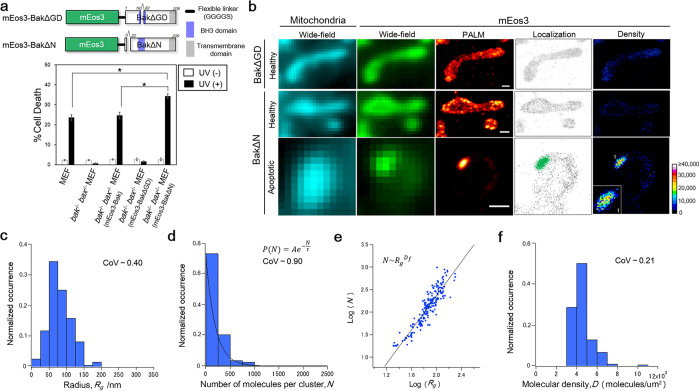
Characterization of mEos3-Bak mutant clusters. (**a**) Schematic constructs of Bak mutants and the trypan blue exclusion assay. Data are represented as mean ± s.e.m. (n = 3). Statistical analysis was performed using one-way analysis of variance (one-way ANOVA) with the Dunnett’s post hoc tests. (**b**) Images of mitochondria (Wide-field) and mEos3 (Wide-field, PALM, Localization and Density) in a healthy cell and an apoptotic cell expressing mEos3-BakΔGD or mEos3-BakΔN. Localizations identified as a cluster are colored (mEos3, Localization). The color bar indicates the molecular density (molecules/μm^2^) in the density maps. A magnified image of a cluster was shown in the inset. Scale bars: 500 nm. (**c**) Normalized histogram of the radii, *R*_*g*_, of mEos3-BakΔN clusters. (**d**) Normalized histogram of the number of mEos3-BakΔN molecules per cluster, *N*. The distribution was fitted by single exponential decay. (**e**) Fractal analysis. Linear regression of the plot yields the slope which corresponds to the fractal dimension, *D*_*f*_. (**f**) Normalized histogram of the molecular densities, *D*, of mEos3-BakΔN clusters. CoV denotes the coefficient of variation obtained for the respective data. N = 195 clusters from five cells.

To further characterize the Bak cluster, we generated two cell lines stably expressing mEos3-labeled Bak mutants: mEos3-BakΔGD (Supplementary Fig. 11) and mEos3-BakΔN (Supplementary Fig. 12). BakΔGD lacks two amino acids in the Bcl-2 homology 3 (BH3) domain which is necessary for the mediation of apoptosis (Supplementary Fig. 1)^21^. The deletion did not abrogate mitochondrial localization in the MEF cells (Supplementary Fig. 11c), but apoptotic function was completely lost (Fig. 5a). Intriguingly, mEos3-BakΔGD formed no cluster under the apoptotic condition (Supplementary Fig. 11c). In addition, a conformational change in BakΔGD, which is requisite for the formation of a functional cluster, was not observed upon UV stimulation (Supplementary Fig. 13a). These results suggest that the deletion in the BH3 domain suppressed the activation of Bak and its subsequent clustering, leading to the loss of apoptotic function. The other Bak mutant, BakΔN, lacks 20 amino acids at the N-terminus (Supplementary Fig. 1). Assays for the viability and caspase-3 activity using *bak*^−/−^ *bax*^−/−^ MEF transiently expressing BakΔN revealed that BakΔN exhibited apoptotic activity higher than that of Bak (Supplementary Fig. 14a). The cell line expressing mEos3-BakΔN showed higher susceptibility to apoptotic stimulation than mEos3-Bak (Fig. 5a), which was consistent with the results of the apoptosis assays based on PARP cleavage (Supplementary Fig. 14b). The mutant localized uniformly on mitochondria in the unstimulated cells and formed clusters upon apoptotic stimulation (Supplementary Fig. 12c,f). The conformational change in BakΔN for its activation during apoptosis was observed from immunostaining analysis (Supplementary Fig. 13b), indicating that mEos3-BakΔN formed functional clusters responsible for apoptosis upon stimulation.

To investigate characteristics of the Bak mutant clusters based on their localization, we observed the BakΔN clusters with PALM at the apoptotic stage (Fig. 5b and Supplementary Fig. 3). Subsequently, we identified mEos3-BakΔN localizations constituting clusters using the cluster algorithm (Supplementary Fig. 7b). Quantitative analysis of the identified BakΔN clusters revealed that the radius (*R*_*g*_) was broadly distributed, 20 nm–199 nm, yielding a mean radius of 82 ± 33 nm (s.d.) (Fig. 5c). The number of molecules per cluster (*N*) was 18–985, yielding a *τ* of 191 molecules (Fig. 5d). The fractal analysis of BakΔN clusters revealed a fractal dimension (*D*_*f*_) of 1.89 ± 0.12 (95% confidence interval) (Fig. 5e). The *D*_*f*_ value of the BakΔN cluster was consistent with that of the wild-type Bak cluster, indicative of a similar density of mEos3-BakΔN molecules within clusters across different sizes. Finally, we measured the molecular density in each BakΔN cluster. The molecular density (*D*), which was calculated by averaging the local densities, exhibited a distribution between 3,154 and 10,906 molecules/μm^2^, resulting in the mean of 4.54 × 10^3^ molecules/μm^2^ (Fig. 5f). The coefficients of variation were 0.40, 0.90, and 0.21, respectively, in *R*_*g*_, *N*, and *D*, indicating that the distribution of *D* was confined to a narrower region than that of *R*_*g*_ and *N*. The homogeneous distribution of the density shows agreement with the scaling relation characterized by *D*_*f*_ . Unlike *R*_*g*_ and *N*, we found no significant difference in *D* between Bak and BakΔN clusters (Supplementary Fig. 15 and Table 1).

## Discussion

We used a PALM-based approach for quantitative characterization of the Bak clusters in terms of the radius and the number of molecules per cluster. Using this approach, we characterized clusters smaller than the diffraction limit (Fig. 4b), which constitutes the vast majority of clusters. In addition, our approach based on single molecule localization provided insight into the number of molecules per cluster. It has already been reported that only 40% of mEos3 in a cell are detectable by PALM because of the existence of misfolded non-fluorescent proteins^22^. However, this underestimation of mEos3 in the present study does not perturb the estimation of the distribution of the number of molecules per cluster because the degree of underestimation is constant among clusters (Fig. 4c). The distribution was approximately exponential without characteristic size, suggesting that Bak proteins spontaneously oligomerized without an additional mechanism such as regulation by other mitochondrial proteins^11^.

Bax, a close homolog of Bak, functions as a similar pro-apoptotic protein via clustering on mitochondria^2^. According to some previous studies^6^,^7^, the number of Bax proteins per cluster varies considerably from 70–250 molecules to 1 × 10^3^–2 × 10^4^ molecules. These values were evaluated with a cell in the presence of endogenous Bax using conventional fluorescence microscopy. To count the Bax proteins, the fluorescence intensity of each cluster was measured. Then the number of fluorescent proteins in each cluster was estimated from a calibration graph using its corresponding fluorescent protein. In contrast to our evaluation of the previous approach, we emphasize that the present approach is more accurate. We replaced endogenous Bak by mEos3-labeled Bak (Fig. 1) and performed *in situ* characterization of Bak clusters without any need for *in vitro* calibration (Fig. 4). Considering the degree of underestimation described above, we estimated that the number of molecules per cluster was from approximately several tens to 6 × 10^3^, whereas 90% of the clusters included fewer than 2 × 10^3^ proteins. Our approach revealed the existence of small clusters comprising several tens of Bak proteins.

The formation of Bak/Bax clusters is primarily responsible for releasing apoptogenic proteins from mitochondria into the cytosol. Despite extensive studies on the mechanism, however, it remains unclear how these clusters permeabilize the mitochondrial outer membrane. Two models have been proposed to explain the mechanism; proteinaceous pore model^23^ and lipidic pore model^24^. In the former model, Bak/Bax clusters themselves serve as a channel-like pore on mitochondrial outer membrane. Alternatively, in the lipidic pore model, the cluster formation induces a mechanical tension of the mitochondrial membrane, thereby forming lipidic pores on the membrane. As discussed above, we found that the Bak clusters consisted of approximately several tens to thousands Bak molecules in the cellular milieu. This cluster size was far larger than a typical channel such as that reconstituted into liposome^23^. Furthermore, the pore-like structure was not observed in the distribution of Bak proteins in each cluster (Fig. 4a, Localization and Density). These results suggest that the Bak cluster does not form a proteinaceous pore but induces a mechanical tension of the membrane, although the possibility of the proteinaceous pore model might still be remained. In addition, we revealed that the molecular density in the Bak clusters was homogeneous among different-size clusters (Fig. 4), which was similar to that in BakΔN clusters (Fig. 5 and Supplementary Fig. 15c). Recently, it has been suggested that several amino acid regions in Bak are shallowly inserted into the mitochondrial outer membrane during its clustering, thereby increasing mechanical tension and curvature in the membrane^25^,^26^. Moreover, biochemical analysis using artificial membranes revealed that the pore size depends on the concentration of Bak proteins on the membrane^27^. Taken together with such previous knowledge, our results suggest that the cells maintained low Bak density (1.55 × 10^3^ molecules/μm^2^, Supplementary Fig. 4d) under unstimulated conditions, thereby avoiding the perturbation of the integrity of mitochondrial membrane. Upon UV stimulation, Bak underwent its conformational change and the density increased locally on mitochondria, which was estimated as 4.75 × 10^3^ molecules/μm^2^ (Table 1). The local Bak clusters with the determinant density might engender mechanical stress on the mitochondrial membrane to form the lipidic pore that enabled apoptogenic proteins to be released from mitochondria to the cytosol.

Recently, two independent groups have reported using super-resolution microscopy that Bax forms several cluster structures in apoptotic cells. The Bax molecules would evolve from linear or arc-shaped structure into a ring-like structure during apoptosis^28^,^29^. In addition, a study using atomic force microscopy showed that these structures of Bax cluster were associated with membrane pores, which suggested that these structures were responsible for the membrane permeabilization^28^. On the other hand, we could not find any ring-like structures in case of Bak cluster even in the apoptotic cells. A possible reason for the differences is that the structure of Bak cluster in apoptotic cells may be different from that of Bax cluster though the function of Bak is similar to Bax. Furthermore, even if Bak would form a ring-like structure in apoptotic cells, insufficient resolution in the present microscope system might hamper observation of the ring-like structure. In fact, fluorescence intensity of mEos3 used in this study is much weaker than that of the Alexa647 used in the recent study^28^, which strongly affects the resolution of the fluorescence images. Further studies using super-resolution microscopy, such as time-lapse imaging of Bak/Bax with high spatial and temporal resolution, will provide insights into the changes in the structure of Bak/Bax cluster on mitochondrial membrane.

In summary, we demonstrated an approach based on single-molecule localization to *in situ* characterization of Bak clusters essential to the induction of apoptosis. Directly visualizing each mEos3-labeled Bak in the physiologically relevant cluster with super-resolution accuracy, we demonstrated the existence of clusters smaller than the diffraction limit and quantitatively characterized their size in the cellular milieu. We provided unprecedented insight into the density of Bak proteins in each cluster, revealing that the density is homogeneous among clusters with divergent sizes. In addition, we could not find any pore-like structure of each Bak cluster on mitochondrial membrane with a resolution of 20 nm. The present approach will provide an exceptional opportunity for elucidation of the mechanism by which the protein cluster enables the mitochondrial membrane to be permeable in the cells.

## Methods

### Cell culture and induction of apoptosis

Wild-type MEF, *bak*^−/−^ *bax*^−/−^ MEF and Plat E cells were cultured in DMEM (high glucose) supplemented with 10% FBS, 100 unit/ml penicillin and 100 μg/ml streptomycin. The cells were incubated at 37 °C in 5% CO_2_ atmosphere. To induce apoptosis, MEFs were irradiated with UV light (254 nm, 100 mJ/cm^2^) using a CL-1000 ultraviolet cross-linker (UVP, Inc., Upland, CA).

### Plasmid construction, viral infection and preparation of stable cell lines

All genes were amplified by PCR using PrimeSTAR DNA polymerase (Takara Bio Inc.) and customized specific primers. The cDNA of Bak was amplified from mouse cDNA library. cDNA of mEos3 (I102N/H158E/Y189A mutant of mEos2) was generated by the site-directed mutagenesis with a template cDNA of mEos2. mEos3-Bak was developed by connecting cDNAs of mEos3 and Bak with a flexible linker (GGGGS), and was subcloned into a retroviral vector, pMX. mEos3-BakΔGD and mEos3-BakΔN were constructed by deleting the corresponding regions from mEos3-Bak (six bases and 60 bases for mEos3-BakΔGD and mEos3-BakΔN, respectively), using KOD Plus Mutagenesis Kit (TOYOBO Co., Japan). For viral infection, the plasmids were transfected into Plat E cells with the TransIT-LT1 reagent (Mirus Co., TX). The supernatants of the media containing the viruses were obtained 48 hours after the transfection, and were added to *bak*^−/−^ *bax*^−/−^ MEF for stable expression. Infected *bak*^−/−^ *bax*^−/−^ MEF cells were cultured in 96-well plates in limiting dilutions to obtain the single clones. Clones that showed comparable morphology and expression level to those of wild-type MEF were selected and used for further experiments.

### Western blot analysis

Cells were collected and treated with a lysis buffer (10 mM Tris-HCl pH 7.4, 150 mM NaCl, 5 mM EDTA, 50 mM NaF 0.5% NP-40). After centrifugation, the supernatants were boiled in a sample buffer (250 mM Tris-HCl pH 6.8, 10% β-mercaptoethanol, 4% SDS, 10% sucrose) for 5 min. The proteins were subjected to SDS-PAGE and were subsequently transferred to a nitrocellulose membrane. The membrane was probed with anti-Bak antibody (Bak NT; Millipore Corp.) and anti-rabbit IgG conjugated with horseradish peroxidase (HRP) (Jackson Immuno Research Laboratories, Inc., West Grove, PA). The proteins were detected using SuperSignal West Femto Maximum Sensitivity Substrate (Thermo Fisher Scientific, Loughborough, UK) and LAS 4000 mini CCD camera (GE Healthcare, Little Chalfont, Buckinghamshire, UK). Subsequently, the membrane was reprobed by anti-β-actin antibody (Sigma) followed by an HRP-conjugated anti-mouse IgG antibody (Jackson Immuno Research Laboratories, Inc., West Grove, PA) for detection of β-actin.

For western blotting to examine PARP cleavage, both alive and dead cells were harvested with PBS and 2.5 g/L Trypsin/1 mmol/L EDTA Solution (nacalai tesque) and centrifugation of samples at 5,000 rpm. The pellets were directly lysed in 2X SDS sample buffer supplemented with proteinase inhibitor cocktail (nacalai tesque). All samples were fractionated by electrophoresis with SDS-polyacrylamide gel plates (BIO GRAFT), and the separated proteins were transferred to a polyvinylidene difluoride membrane (Millipore Corp.). The membrane was incubated for 60 min at room temperature with PBS containing 0.5% Tween 20 and 3% dried skim milk (nacalai tesque) before exposure to primary antibodies to PARP1 (Cell Signaling Technology) or to tubulin (Thermo Fisher Scientific). Immune complexes were detected with alkaline phosphatase- or horseradish peroxidase-conjugated secondary antibodies (Cell Signaling Technology) at a dilution of 1:5000 and Chemi-Lumi One Super reagent (nacalai tesque).

### Quantification of apoptosis

MEFs were seeded in 35 mm dishes at a cellular density of 1.0 × 10^5 ^cells/ml. After 24 hours, the cells were stimulated with UV light. The cells and medium were collected 24 hours after UV stimulation, and then centrifuged for 2 min at 600 *g* at room temperature. After supernatant was removed, 200 μl of DMEM was added to the pellet. The pellet was suspended by gentle pipetting. Then 200 μl of 0.4% Trypan Blue Solution (Wako Pure Chemical Inds. Ltd.) was added. After 1 min incubation, the samples were subjected to an automatic cell counter (Countess, Invitrogen Corp.) to measure the percentages of cell death. Activities of caspase-3 were measured with DEVD-MCA reagent (Peptide Institute, Inc.) according to the manufacturer’s instruction.

### Immunostaining

After UV irradiation as an apoptotic stimulation and treatment with Mitotracker Red CMXRos (Invitrogen Corp.) to stain mitochondria, cells on a cover glass were fixed with 4% paraformaldehyde (Alfa Aesar, USA). The cells were permeabilized with 0.2% Triton X-100 in PBS and then blocked by 0.2% fish skin gelatin (FSG) in PBS. The buffer was exchanged to 0.2% FSG in PBS containing mouse anti-Bak antibody (Abcam) or rabbit anti-Bak antibody (Bak NT) and incubated overnight at 4 °C, shaking gently. After washing with PBS, the cells were incubated with 0.2% FSG in PBS containing a donkey anti-mouse antibody conjugated with Alexa488 (Molecular Probes, Inc.) or a donkey anti-rabbit antibody conjugated with Alexa647 (Molecular Probes, Inc.). The cells were washed with 0.2% FSG in PBS and were mounted on a slide glass with FluorSave Reagent (Calbiochem). Fluorescence images of the samples were acquired using the laser scanning confocal microscope.

### PALM imaging

After UV stimulation, cells on a cover glass were treated with Mitotracker Deep Red FM (Invitrogen Corp.) and fixed with 4% paraformaldehyde in phenol red free DMEM at 37 °C for 10 min. The cells were washed with PBS and imaged using a home-built TIRF microscope constructed on an inverted microscope (IX81; Olympus Corp.) equipped with a PlanApo 100×, 1.49 N.A. oil immersion objective (Olympus Corp.). Four laser lines with 405 nm (JUNO 405; SOC Corp.), 488 nm (CYAN-488; Spectra-Physics), 561 nm (JUNO 561; SOC Corp.), and 640 nm (CUBE 640-100C; Coherent Inc.) were combined in an external platform. Samples were illuminated in highly inclined and laminated optical sheet (HILO) configuration^30^. Fluorescence emission was detected with an EM-CCD camera (ImagEM; Hamamatsu Photonics KK) with an image pixel size of 160 nm. Approximately 22,000 frames were acquired with an exposure time of 30 ms per frame. The number of frames acquired was dependent on the regions of highest mEos3 density. mEos3 was activated with 405 nm laser intensity, which was intermittently increased during a PALM experiment from 1 mW/cm^2^ to 1 W/cm^2^ so that the PALM acquisition maintained a low density of photoactivated fluorophores in each frame. Simultaneously, mEos3 was excited with 561 nm laser intensity of approximately 1 kW/cm^2^. The fluorescence signal from mEos3 was directed to the EM-CCD camera using a polychromatic dichroic mirror (Di01-R405/488/561/635, Semrock Inc.)and a band pass filter (FF01-609/54, Semrock Inc.). The detected signals were localized with PeakSelector software (IDL, courtesy of Harald Hess) using a threshold of 100 photons which were obtained by observing a non-transfected fixed *bak*^−/−^ *bax*^−/−^ MEF. Each fluorescence peak was fitted to a two-dimensional Gaussian distribution by nonlinear least-square fitting to obtain *x* and *y* coordinates. The localization precision for each molecule was experimentally calculated as described previously^31^.

In the PALM experiment, it is highly probable that a single fluorophore emits photons over multiple consecutive frames before being bleached, leading to an overcounting error (Supplementary Fig. 9a,b). Therefore, it is prerequisite to correct the error by grouping the fluorescence signals localized within the specific spatial threshold into the single molecule. In this grouping analysis, fluorescent molecules localized within 64 nm in consecutive frames were counted as the same molecule. The spatial threshold (64 nm), which corresponds to three times the measured mean localization precision, provides the best assignment^32^. In addition to the grouping analysis using the spatial threshold, a correction of another overcounting error caused by blinking of the fluorophore must be conducted for an estimation of the number of molecules. To address this issue, we plotted the number of localizations as a function of a temporal threshold in grouping analysis by observing *bak*^−/−^ *bax*^−/−^ MEF transiently expressing mEos3-Bak (Supplementary Fig. 9c). The plot within the first 1.5 s was fitted by a distribution given by *P* (*t*) = *A*(1 + *n*_*blink*_ *e*^*−t/toff*^), where *n*_*blink*_ is the mean number of blinking events per molecule and *t*_*off*_ is the time that the molecule spends in the dark state^33^. The number of Bak molecules per cluster was obtained by dividing the number of localizations in each cluster by a factor of 1.42, which corresponds to 1 + *n*_*blink*_. A decimal place of each obtained number was omitted. Grouping analysis using a fixed temporal threshold yields a significantly large error whereas the normalization by 1 + *n*_*blink*_ provides a good estimation of number of molecules^34^. PALM images were generated by superimposing the position coordinates of the detected molecules with a width corresponding to their localization precision using the Octane plug-in for ImageJ software^35^.

### Statistical analysis

Statistical analyses were conducted using the two-tailed unpaired Student’s *t*-test, the Mann-Whitney test or one-way analysis of variance (one-way ANOVA) with Dunnett’s post hoc tests. In these analyses, *P* > 0.05 is indicated as not significant (n.s.). Statistically significant *P* values are denoted by an asterisk, i.e., *P < 0.05 and **P < 0.0001.

## Additional Information

**How to cite this article**: Nasu, Y. *et al*. * In Situ* Characterization of Bak Clusters Responsible for Cell Death Using Single Molecule Localization Microscopy. *Sci. Rep*. **6**, 27505; doi: 10.1038/srep27505 (2016).

## Supplementary Material

Supplementary Information

## Figures and Tables

**Table 1:** Summary of characteristics of the Bak and Bak-mutant cluster.

Construct	Radius	Number of molecules	Scaling relation	Molecular density	Analyzed clusters
	*R*_*g*_ (nm)	*τ* (molecules)	*D*_*f*_	*D* (molecules/μm^2^)	
mEos3-Bak	111 ± 50	344 ± 24	1.80 ± 0.09	(4.75 ± 0.06) × 10^3^	433 (4 cells)
mEos3-BakΔGD	No clusters detected.				
mEos3-BakΔN	82 ± 33	191 ± 5	1.89 ± 0.12	(4.54 ± 0.07) × 10^3^	195 (5 cells)

Radii are represented as mean ± s.d. Densities are represented as mean ± s.e.m. The errors of *τ* and *D*_*f*_ are the 95% confidence interval obtained from the regression analyses.
