# Supplementary material for: In Situ Characterization of Bak Clusters Responsible for Cell Death Using Single Molecule Localization Microscopy
Source: Sci Rep. 2016 Jun 13;6:27505. doi: 10.1038/srep27505 (PMC4904369; doi:10.1038/srep27505)
Supplement: Supplementary Information [file srep27505-s1.pdf]

Supplementary Information for

In Situ Characterization of Bak Clusters Responsible for Cell Death Using Single Molecule Localization Microscopy

Yusuke Nasu[1], Alexander Benke[2], Satoko Arakawa[3], Go J. Yoshida[3], Genki Kawamura[1], Suliana Manley[2], Shigeomi Shimizu[3], Takeaki Ozawa[1,*]

[1]Department of Chemistry, School of Science, The University of Tokyo, 7-3-1 Hongo, Bunkyo-ku, Tokyo 113-0033, Japan

[2]Laboratory of Experimental Biophysics, École Polytechnique Fédérale de Lausanne, CH-1015 Lausanne, Switzerland

[3]Department of Pathological Cell Biology, Medical Research Institute, Tokyo Medical and Dental University, 1-5-45 Yushima, Bunkyo-ku, Tokyo 103-5802, Japan

[*]To whom correspondence should be addressed: T. O.

E-mail: ozawa@chem.s.u-tokyo.ac.jp

Supplementary Methods

Live-cell imaging and data analysis. $bak^{-/-}$ $bax^{-/-}$ MEF stably expressing GFP-Bak and Smac-SNAP, which was generated by viral infection, was treated with 3 μM SNAP-Cell TMR-STAR (New England BioLabs Inc.). The cells were washed with DMEM and were then placed in an observation medium (HEPES-buffered DMEM phenol red free, 10% FBS, pH 7.2). After UV irradiation as an apoptotic stimulation, fluorescence time-lapse images of the cells were acquired every 5 min using a laser scanning confocal microscope (IX81-FV1000-D; Olympus Corp.) equipped with UPlanSApo 100×, 1.40 N.A. oil immersion objective. The cells on the stage were kept at 37°C using a stage incubator (Tokai Hit Corp.). The images were analyzed using ImageJ software by drawing regions around the cell and by calculating the standard deviation (SD) of pixel intensities in the regions. The Bak clustering index and Smac release index were calculated by dividing SD by the mean fluorescence intensities of GFP and TMR, respectively.

$bak^{-/-}$ $bax^{-/-}$ MEF stably expressing mEos3-Bak and mCherry-Bax or mCherry-Drp1 were placed in an observation medium (HEPES-buffered DMEM phenol red free, 10% FBS, pH 7.2). After UV irradiation as an apoptotic stimulation, fluorescence images of the cells were acquired using a laser scanning confocal microscope (IX81-FV1000-D; Olympus Corp.) equipped with UPlanSApo 100×, 1.40 N.A. oil immersion objective.

Cluster analysis. To quantify the degree of Bak clustering relative to homogeneous distribution, the spatial point pattern of Bak distribution obtained by PALM was used to calculate Ripley's K-function with SpPack[36, 37]. Ripley's K-function is given by,

$$K(r) = A\sum_{i=1}^{n}\sum_{j=1}^{n}\left(\frac{\delta_{ij}}{n^2}\right) \quad \text{where } \delta_{ij} = 1 \text{ if } \delta_{ij} < r\text{, otherwise } 0. \qquad \textbf{[S1]}$$

Therein, A represents the area of the analyzed region, n stands for the number of points, r denotes the analyzed spatial scale and δ_{ij} is the distance between two points i and j. $K(r)$ represents the normalized number of points encircled by concentric circle with radius r centered on each point. This function, which scales with circle area, was then transformed into the L-function such that the scaling is linear with radius r.

$$L(r) = \sqrt{\frac{K(r)}{\pi}} \qquad \textbf{[S2]}$$

Here, $L(r)$ equals to r at all r for completely random distribution of points. We therefore plotted $L(r) - r$ as a function of r such that a random distribution results in $L(r) - r = 0$ for all r. $L(r) - r$ has positive values for a given r at which the distribution is more clustered than a random distribution. Points at the edge of the analyzed region were weighted to negate edge-related effects[37]. We calculated 99% confidence intervals by simulating 500 spatially random

distributions with the same mean molecular density as the data region.

To objectively identify Bak clusters, we performed cluster analysis using an existing algorithm, *density-based spatial clustering of application with noise* (DBSCAN). DBSCAN is often used for data mining and spatial pattern analysis[14, 18]. Identification of Bak clusters with DBSCAN is based on the density distribution of Bak molecules in a dataset obtained in a PALM experiment. Bak localizations with greater than *MinPts* neighbors within a radius ε are connected to their neighbors. These connected localizations are regarded as clustered. Bak localizations that are not connected to any other localizations are regarded as be non-clustered noise.

There are two parameters in DBSCAN: ε and *MinPts*. Because the mean molecular densities in healthy cells were comparable in mEos3-Bak and mEos3-BakΔN (Supplementary Fig. 4d), it was possible to meaningfully use the same values of the parameters for these datasets. We chose ε of 20 nm, which corresponds to the mean localization precision. *MinPts* should be sufficiently large to separate them from noise, but it should not be too large as to overlook a part of clusters. We determined *MinPts* based on datasets in healthy cells which were not considered to contain any physiological Bak clusters and found that *MinPts* = 13 provided the best result in this regard. Finally, we defined the localizations identified with DBSCAN as a cluster containing a minimum number of 18 Bak molecules, which was previously shown with a biochemical study[3].

Cluster characterization. After identifying Bak clusters with DBSCAN, we examined a set of different parameters to characterize Bak clusters. The following quantitative characterizations were performed using the list of Bak localization coordinates and not the rendered PALM images[38]. The radius of each cluster, R_g, was calculated as the radius of gyration of all localizations within the cluster.

$$R_g = \sqrt{\frac{1}{N}\sum_{i=1}^{N}(\mathbf{r_i}-\mathbf{r_c})^2} = \sqrt{\frac{1}{N}\sum_{i=1}^{N}\left\{(x_i-x_c)^2+(y_i-y_c)^2\right\}} \quad \textbf{[S3]}$$

Therein, N stands for the number of localizations belonging to each cluster, $\mathbf{r_i}$ (x_i, y_i) is the coordinate of each localization within a cluster, and $\mathbf{r_c}$ (x_c, y_c) is the coordinate of the center of mass. The measurement of R_g is based on two-dimensional (2D) projection of three-dimensional (3D) cluster. The relationship between R_g and radius of 3D cluster, R, is given by,

$$R = \sqrt{\frac{1}{N}\sum_{i=1}^{N}\left\{(x_i-x_c)^2+(y_i-y_c)^2+(z_i-z_c)^2\right\}} = R_g\sqrt{1+\frac{\sum_{i=1}^{N}(z_i-z_c)^2}{\sum_{i=1}^{N}(x_i-x_c)^2+\sum_{i=1}^{N}(y_i-y_c)^2}} \quad \textbf{[S4]}$$

where z_i and z_c represent the coordinate along z axis of each localization and the center of mass, respectively. Eq. **S4** shows the degree of underestimation in the measurement of cluster radius

attributable to the 2D projection. Assuming that the variances of cluster size along the x, y, and z axes are comparable, we underestimate the radius approximately by 20%.

The fractal dimension, D_f, was defined as

$$N = k\left(\frac{R_g}{a}\right)^{D_f} = \left(\frac{k}{a^{D_f}}\right)R_g^{\ D_f}, \qquad \textbf{[S5]}$$

where N denotes the number of localizations belonging to each cluster, a represents the radius of a Bak molecule, and k is the structural coefficient[19]. The scattered plot of N with R_g in logarithmic fashion was fitted by a linear model to obtain the slope, which corresponds to D_f.

Local molecular densities were measured by counting the number of localizations within a square with a side of 20 nm.

Correlative microscopy. Cells expressing mEos3-Bak were grown on a grided glass-bottom dish for 24 hours before fixation. After UV irradiation, cells were fixed with a buffer (1.5% paraformaldehyde and 3% glutaraldehyde in 0.1 M phosphate buffer, pH 7.3) for 15 min at room temperature. The cells were washed with distilled water and set under the TIRF microscope for PALM imaging in which the acquisition procedure was virtually identical to the protocol described above. The grid number, where a cell formed Bak clusters, was registered in PALM imaging for the identification of cells in the subsequent electron microscopy. After PALM imaging, electron microscopy was performed as described previously[39]. Briefly, the cells observed in PALM were fixed by an aqueous solution of 1% OsO_4 for 30 min on ice. The sample was embedded in Epon 812 and thin sections (70–80 nm) were then cut. Each section was stained with uranyl acetate and lead citrate for observation under an electron microscope (JEM-1010; JEOL) at 80 kV.

STED imaging. *bak*$^{-/-}$ *bax*$^{-/-}$ MEF stably expressing SNAP-Bak, which was generated by viral infection, was treated with 3 μM SNAP-Cell TMR-STAR. The cells were washed with DMEM and were then placed in an observation medium (Leibovitz’s L-15 medium, phenol red free; Thermo Fisher Scientific). After UV irradiation as an apoptotic stimulation, fluorescence images of the cells were acquired using STED microscope (Leica TCS SP8 STED 3X; Leica Microsystems) equipped with HC PL APO CS2 100×, 1.40 N.A. oil immersion objective. Laser lines with 552 nm and 660 nm were used as excitation and STED light, respectively.

Supplementary Figures

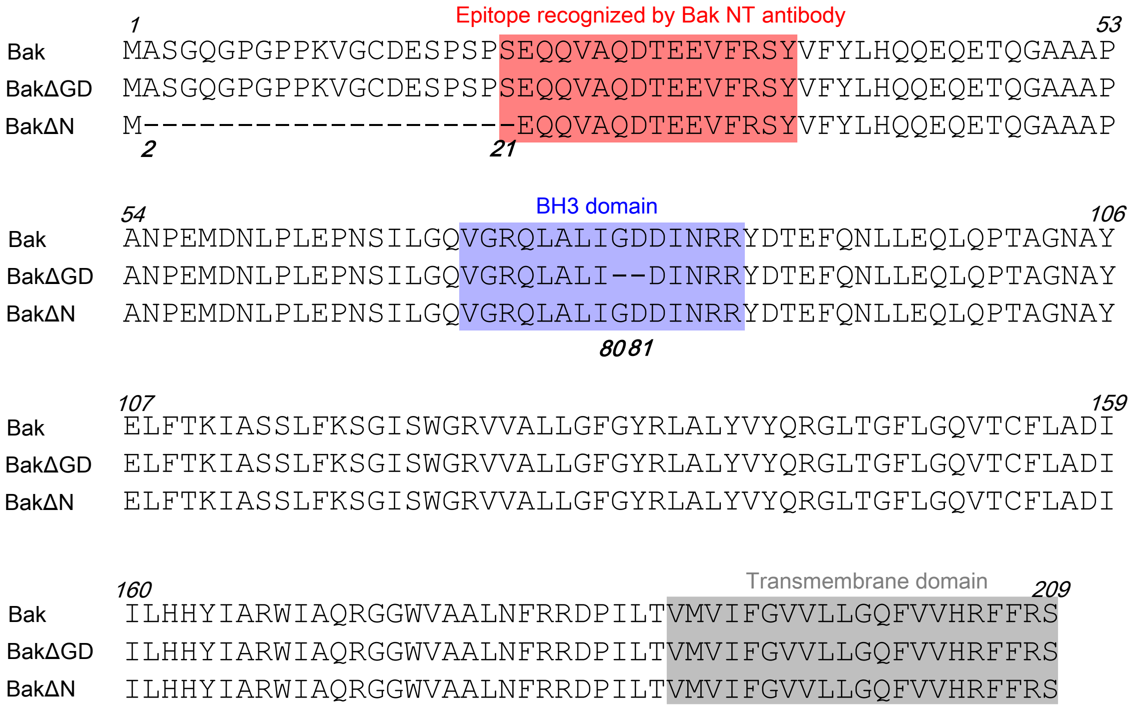

Supplementary Figure 1. Sequences of Bak mutants. Mouse Bak (wild type) is composed of 209 amino acids. Two (^{80}G^{81}D) and twenty (from ^{2}A to ^{21}S) amino-acid residues were deleted in BakΔGD and BakΔN, respectively. The sequence highlighted in the red box corresponds to the epitope recognized by Bak NT antibody. The deletion in BakΔGD is located in a BH3 domain, which is requisite for the induction of apoptosis[21]. The transmembrane domain indicated by gray box is anchored to the mitochondrial outer membrane, thereby allowing Bak to localize in mitochondria[3].

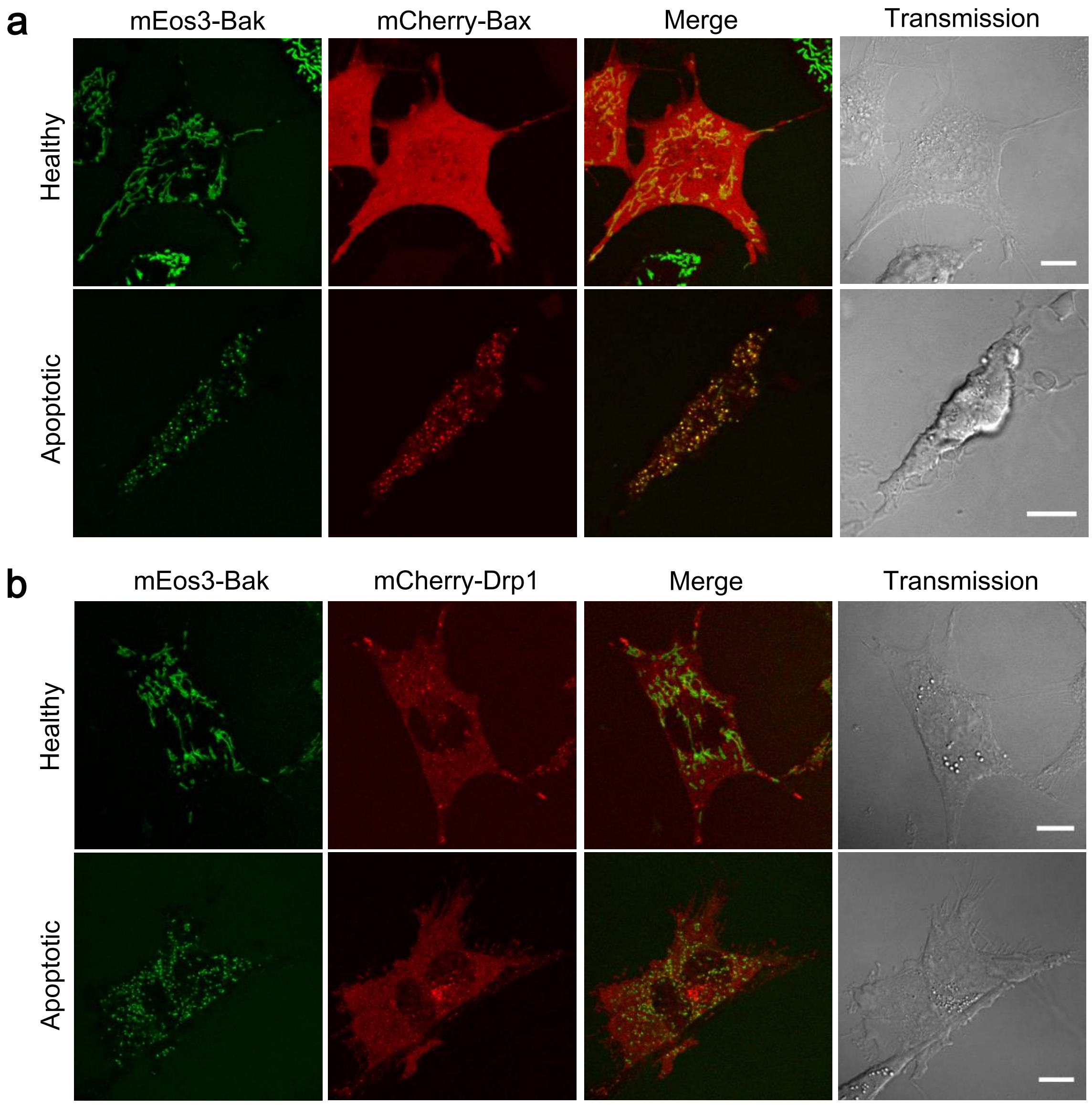

Supplementary Figure 2. Localization of Bak cluster and Bax/Drp1 cluster. (a) Confocal imaging of the cells expressing mEos3-Bak and mCherry-Bax in the presence or absence of UV stimulation. (b) Confocal imaging of the cells expressing mEos3-Bak and mCherry-Drp1 in the presence or absence of UV stimulation. Scale bars: 10 µm.

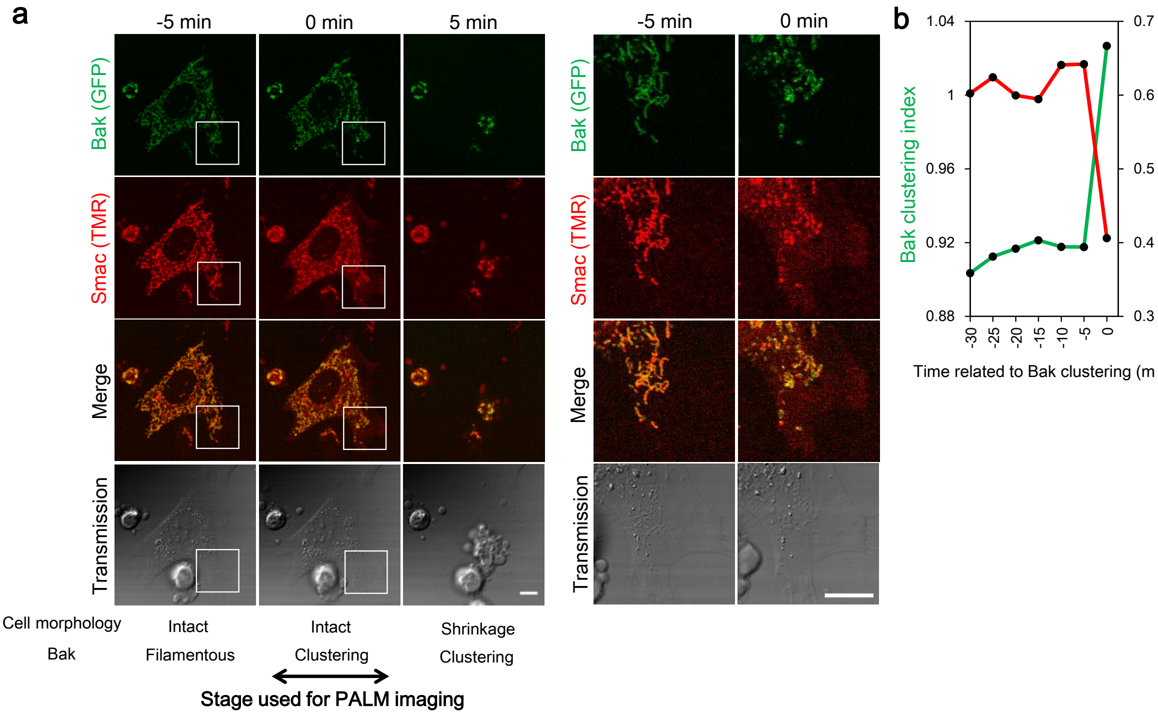

Supplementary Figure 3. Time-lapse imaging of the formation of Bak clusters and the release of Smac during apoptosis. (a) Dynamics of Bak and pro-apoptotic protein Smac in a UV-stimulated *bak*$^{-/-}$ *bax*$^{-/-}$ MEF stably expressing GFP-Bak and Smac-SNAP. Magnified images in the white boxes are shown (right). Punctate localization of GFP-Bak and cytosolic localization of Smac-SNAP at time 0 indicate the formation of Bak clusters and the release of Smac from mitochondria, respectively. The stage, at which cell morphology remained intact but Bak clusters were formed, was used for PALM imaging. Scale bars: 5 μm. (b) Quantitative analysis of the dynamics of Bak and Smac presented in (a).

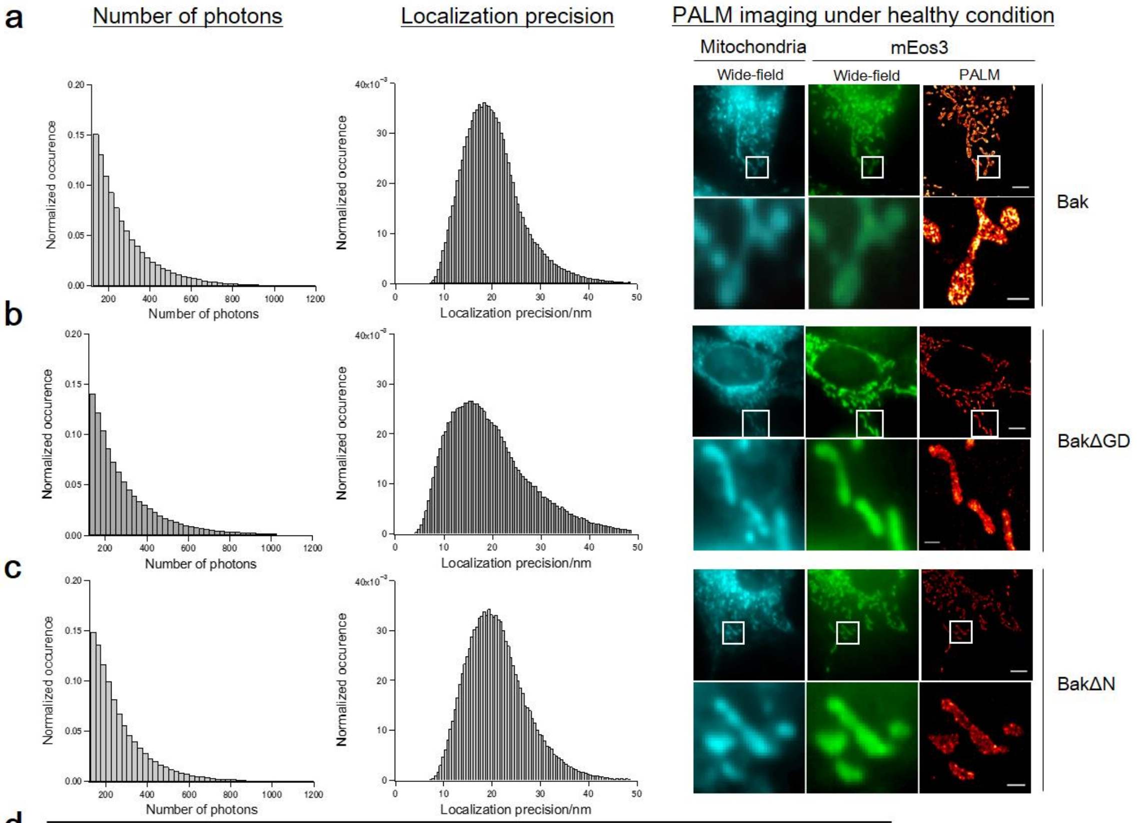

d

Construct	Number of photons	Localization precision (nm)	Density (molecules/μm^2)
mEos3-Bak	256	19.6 ± 6.2	(1.55 ± 0.23) × 10^3
mEos3-BakΔGD	270	18.9 ± 8.4	(1.88 ± 0.51) × 10^3
mEos3-BakΔN	244	20.5 ± 6.4	(1.53 ± 0.33) × 10^3

Supplementary Figure 4. Localization precision and molecular density. Distribution of total photon numbers per burst and localized precision in the engineered cell lines expressing (a) mEos3-Bak, (b) mEos3-BakΔGD, and (c) mEos3-BakΔN. For the analyses, fluorescence emission with less than 100 photons was discarded as background noise and hence not shown in the distribution. PALM images of each cell line under a healthy condition used for the analyses are shown (right). Images of the boxed regions are magnified in the lower panels. Scale bars: 5 μm (top), 1 μm (bottom). (d) Summary of the localization precision and the molecular density of mEos3-Bak, mEos3-BakΔGD and mEos3-BakΔN. Molecular densities were measured in healthy cells where Bak homogeneously distributed on mitochondria. Data are represented as mean ± s.d.

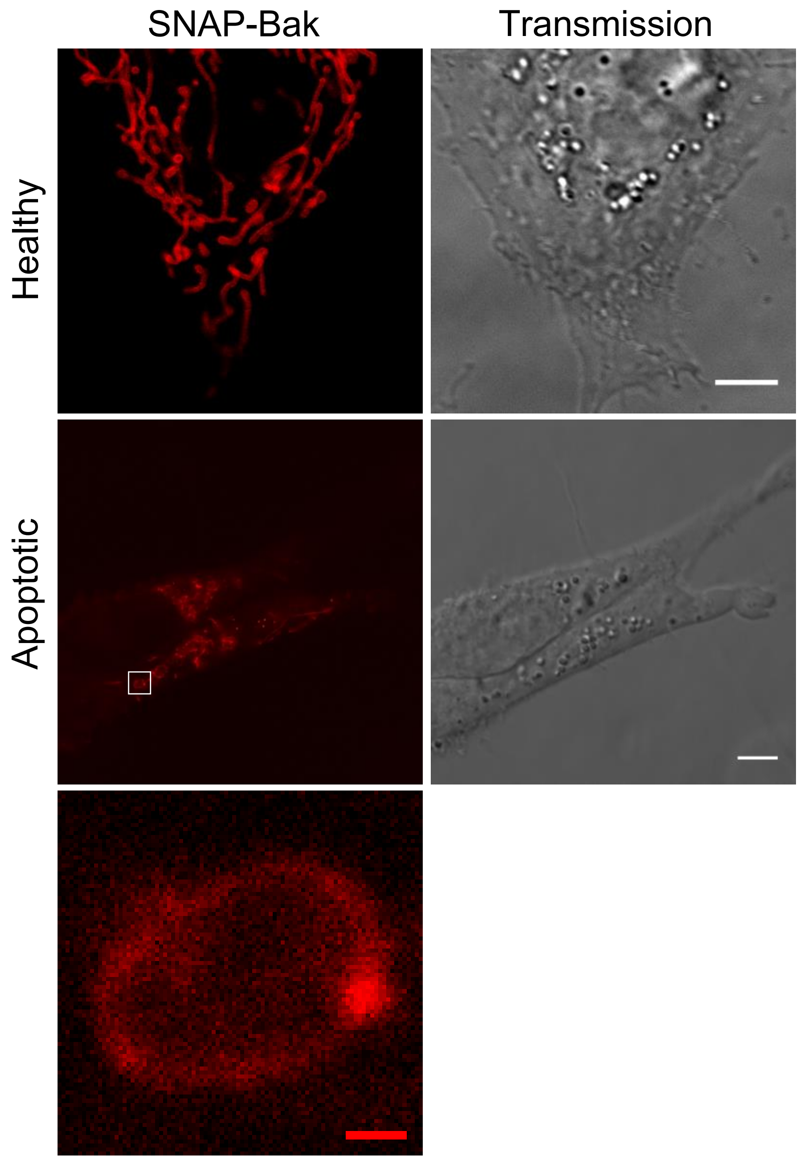

Supplementary Figure 5. Live-cell super-resolution imaging of Bak cluster. Magnified image in white box (middle) is shown at the bottom. Scale bars: 5 µm (Transmission) or 300 nm (Magnified).

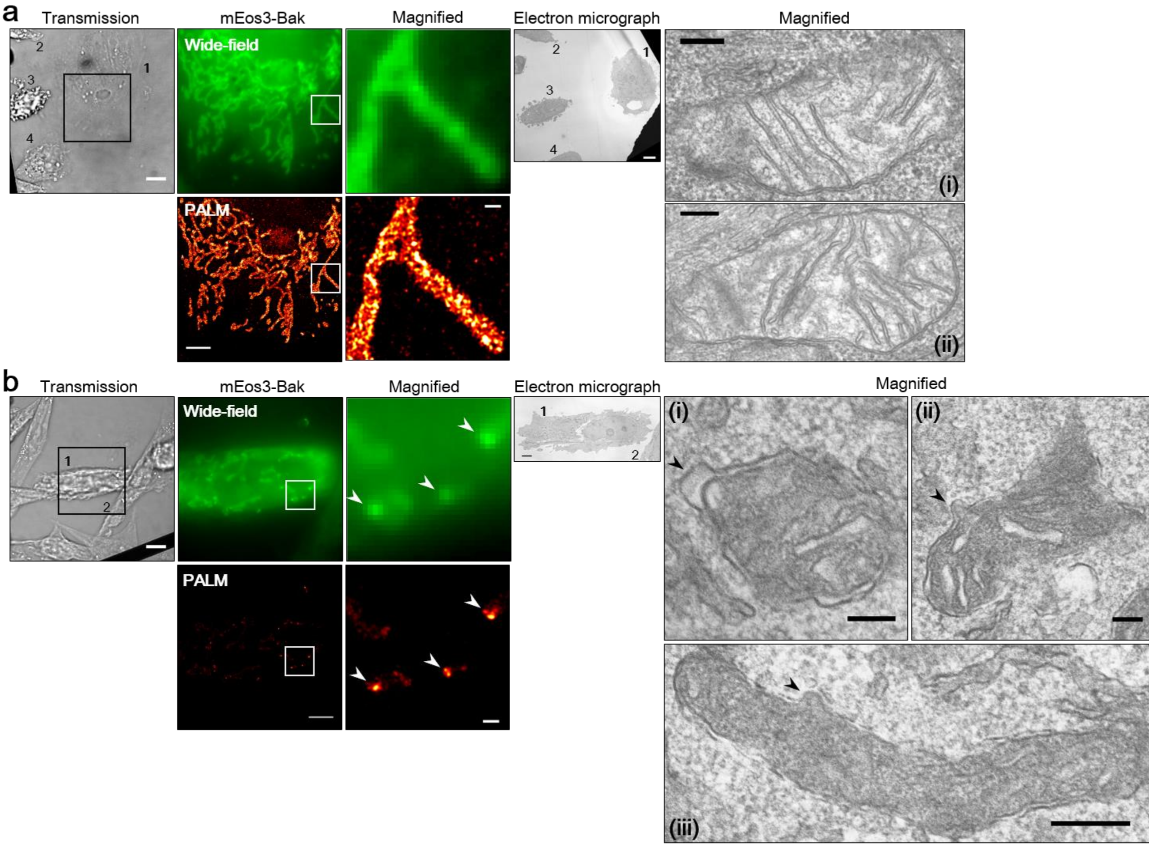

Supplementary Figure 6. Correlative microscopy. (a) PALM and electron microscopy (EM) micrograph in the cell line expressing mEos3-Bak under unstimulated conditions. The region in the black square corresponding to cell **1** was imaged by PALM. The regions in white squares were magnified, showing a filamentous localization of mEos3-Bak. A low magnification electron micrograph represented the same field shown in the optical transmission image. Cell **1**, imaged by EM with higher magnification, showed a clear cristae structure (i and ii). Scale bars: 10 µm (Transmission), 3 µm (PALM), 500 nm (PALM, magnified), 5 µm (EM, low magnification), or 200 nm (EM, high magnification). (b) PALM and EM micrograph in an apoptotic cell. Cells stimulated with UV were imaged by PALM. Subsequently, EM images were acquired in the same cell (cell **1**). White and black arrowheads indicate the formation of Bak clusters in PALM and the distorted membrane structures in EM (i, ii, and iii), respectively. Scale bars: 10 µm (Transmission), 3 µm (PALM), 500 nm (PALM, magnified), 3 µm (EM, low magnification), 200 nm (EM, high magnification), or 20 nm (EM, inset).

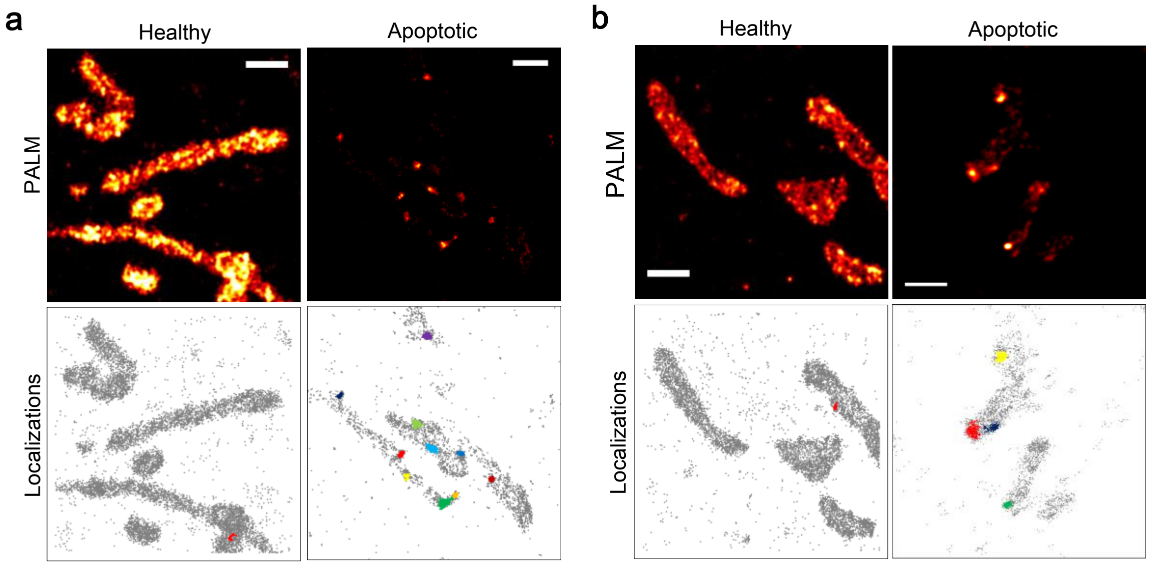

Supplementary Figure 7. Cluster analysis to identify Bak clusters using DBSCAN. PALM images and Bak localizations plotted as gray points in the cell lines expressing (a) mEos3-Bak and (b) mEos3-BakΔN. Localizations defined as a cluster by DBSCAN share the same randomly chosen color. Scale bars: 1 μm.

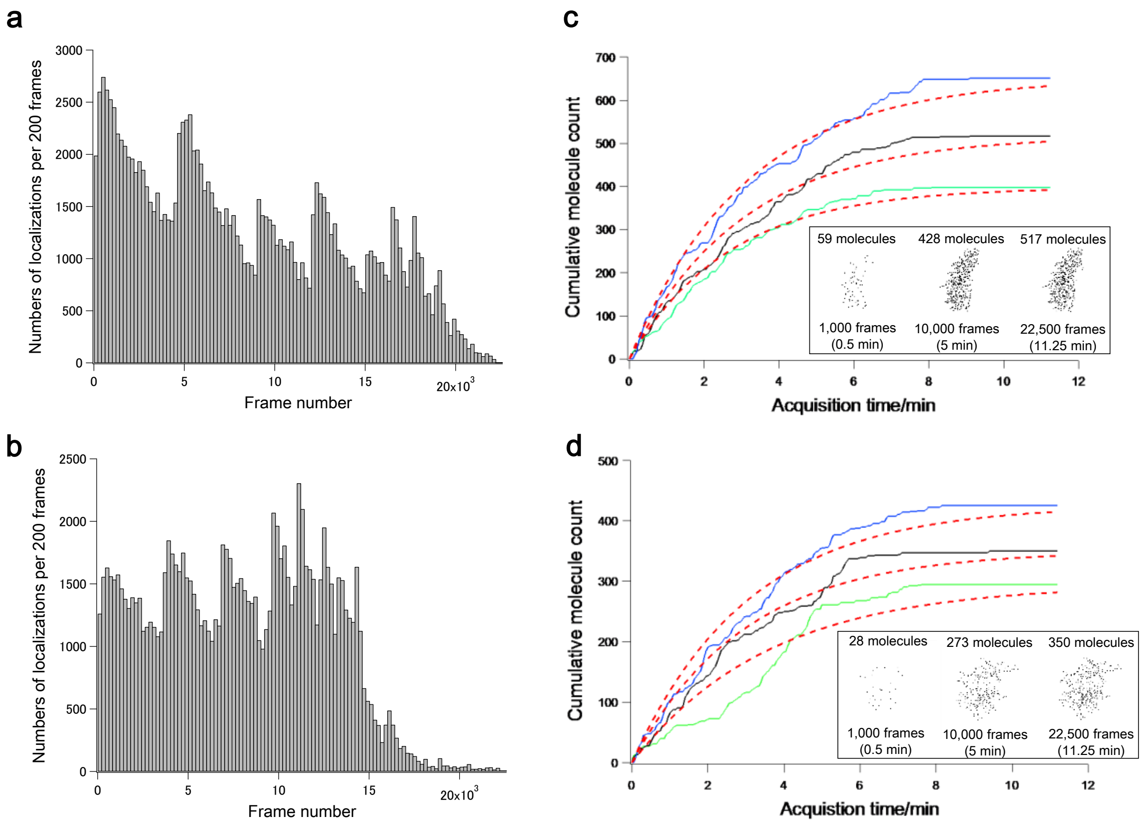

Supplementary Figure 8. Complete PALM imaging and molecular counting in a cluster. The number of localizations per 200 frames was plotted as a function of the frame number in a representative PALM imaging of (a) mEos3-Bak and (b) mEos3-BakΔN. Occasional increases in the intensity of violet light used for photoactivation resulted in an increase in the detected localizations. At the end of the experiment (frame number 22,500), no more fluorescence signal of mEos3-Bak or mEos3-BakΔN was detected, indicating a stoichiometric read-out. Cumulative molecular count for three representative (c) mEos3-Bak clusters and (d) mEos3-BakΔN clusters as a function of acquisition time. The plots, which finally reached a plateau, were fitted to an exponential function (dashed red lines)[12]. The images after 0.5 min, 5 min and 11.25 min of the cluster plotted as a black line in the graph are shown in the inset.

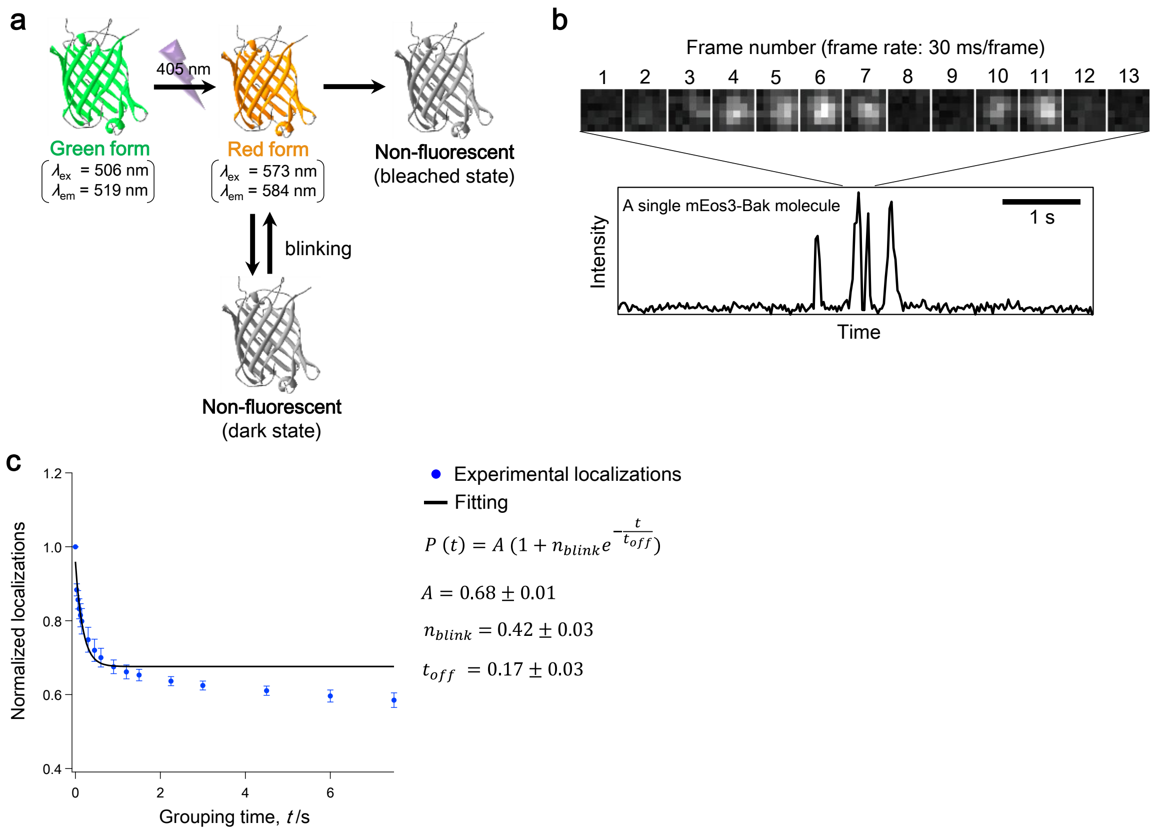

Supplementary Figure 9. Measurement of blinking events of mEos3. (a) Schematics of the fluorescence property of mEos3. Green form of mEos3 is irreversibly converted to the red form upon violet light illumination. In the presence of excitation light, the red form emits fluorescence and converts either to a dark state or to a bleached state. Once the molecule is bleached, no fluorescence signal is detected. In contrast, if the red form of mEos3 converts to another non-fluorescent state (dark state), the fluorescence signal is detected by returning to the red form. This phenomenon is observed as blinking. (b) Time trace of fluorescence intensity of a photoactivated red form of a single mEos3-Bak molecule in *bak*$^{-/-}$ *bax*$^{-/-}$ MEF transiently expressing mEos3-Bak. The trace showed that a single mEos3 molecule blinked several times. Its fluorescence signal was detected over multiple consecutive frames before being bleached. The upper part presents the fluorescence images of a single mEos3-Bak molecule detected in the EM-CCD camera. (c) Number of localizations as a function of grouping time, *t*, in *bak*$^{-/-}$ *bax*$^{-/-}$ MEF transiently transfected with *mEos3-Bak*. Data measured within the first 1.5 s were fitted by a distribution given by $P(t) = A(1+n_{blink}\, e^{-t/t_{off}})$, where n_{blink} is the mean number of blinking events per molecule and t_{off} denotes the time that the molecule spends in the dark state[33]. Data are represented as mean ± s.d. (n = 3 cells).

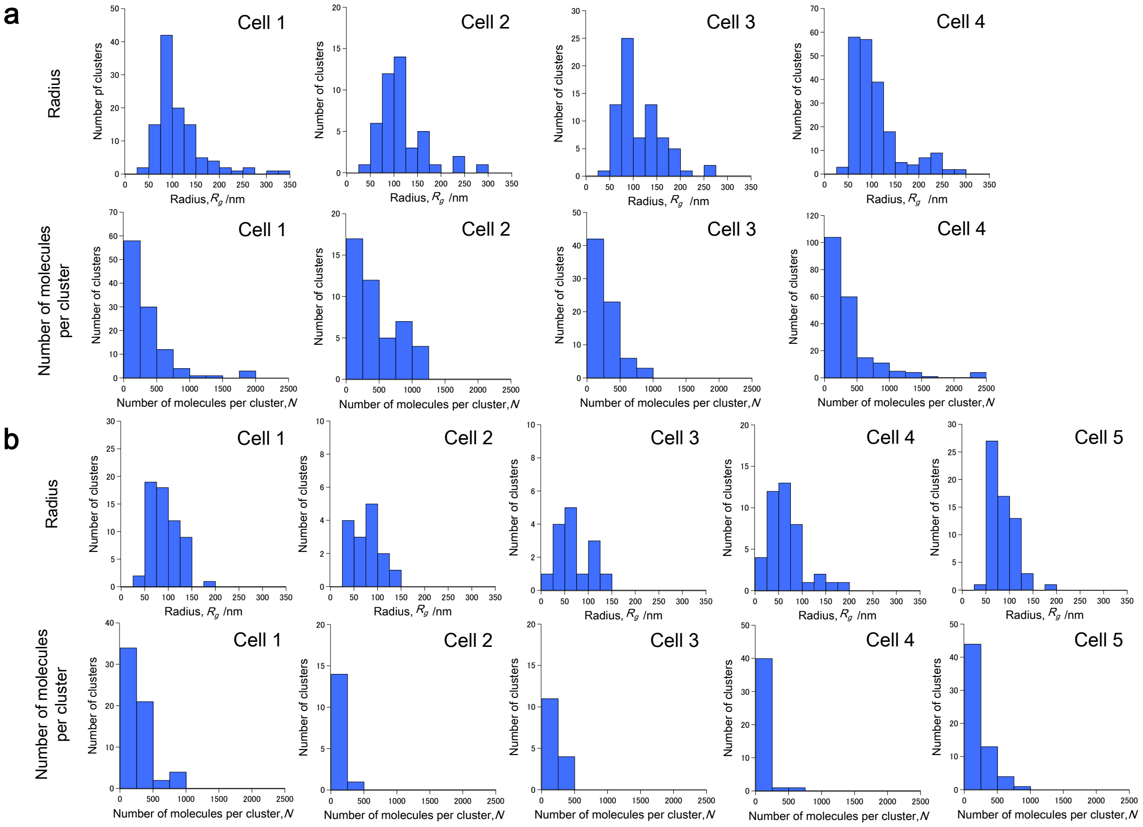

Supplementary Figure 10. Distribution of the radius and number of molecules per cluster in each cell. Distribution of radius (R_g) and number of molecules per cluster (N) of (a) mEos3-Bak clusters and (b) mEos3-BakΔN clusters in each cell.

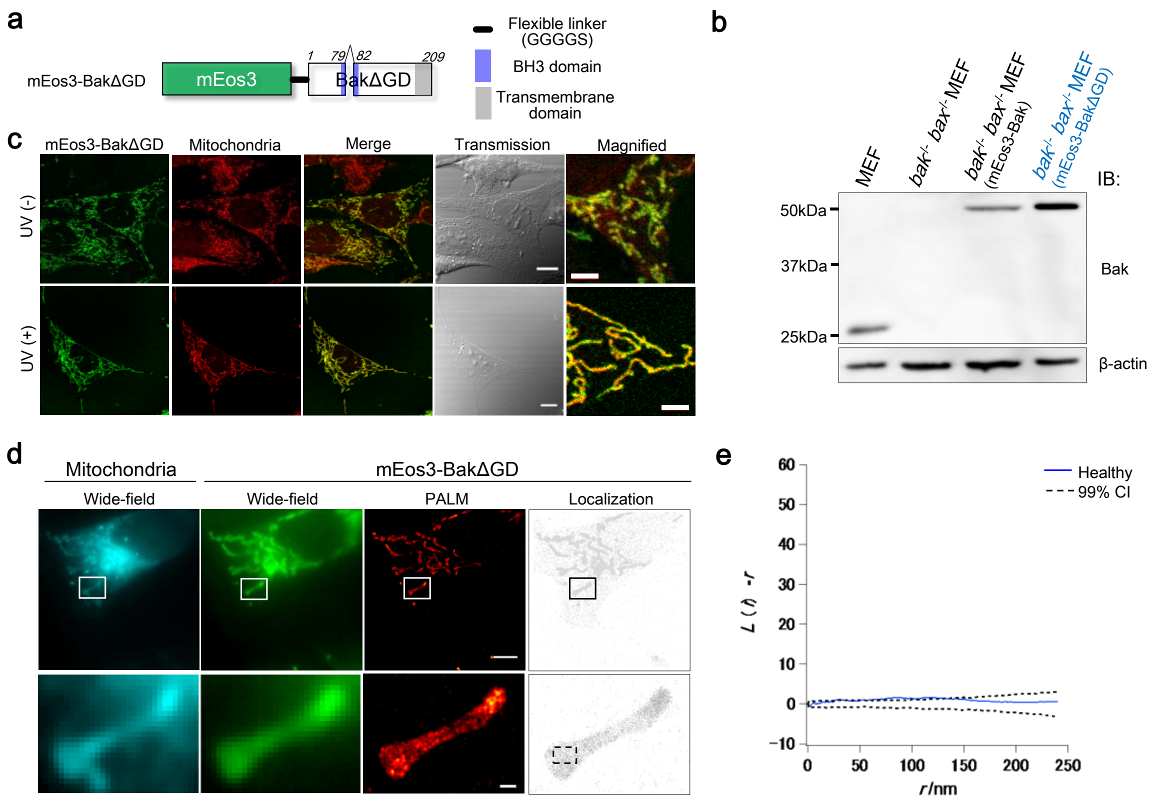

Supplementary Figure 11. Generation of a cell line expressing mEos3-BakΔGD for PALM. (a) Schematic construct of mEos3-BakΔGD. Numbers in italics show the residue of amino acids in Bak. BakΔGD mutant lacks two amino acids (Gly at 80 and Asp at 81), which were located in the BH3 domain (72–86, blue). (b) Western blot analysis for investigating the expression of mEos3-BakΔGD in the engineered cell line. (c) Fluorescence and transmission images of the cells in the presence or absence of UV irradiation. A dye of MitoTracker CMXRos was used for mitochondrial visualization. Scale bars: 10 μm (Transmission) or 2 μm (Magnified). (d) PALM imaging of BakΔGD in a healthy cell. Mitochondria were visualized with MitoTracker Deep Red dye under a wide-field configuration. The boxed regions are enlarged in lower panels. Scale bars: 5 μm (top) or 500 nm (bottom). (e) Ripley's K analysis for quantitative measurement of the degree of the spatial distribution of mEos3-BakΔGD under a healthy condition. The analysis was performed in the boxed region (480 nm × 800 nm, dotted line) of a healthy cell shown in (d). CI, confidence interval.

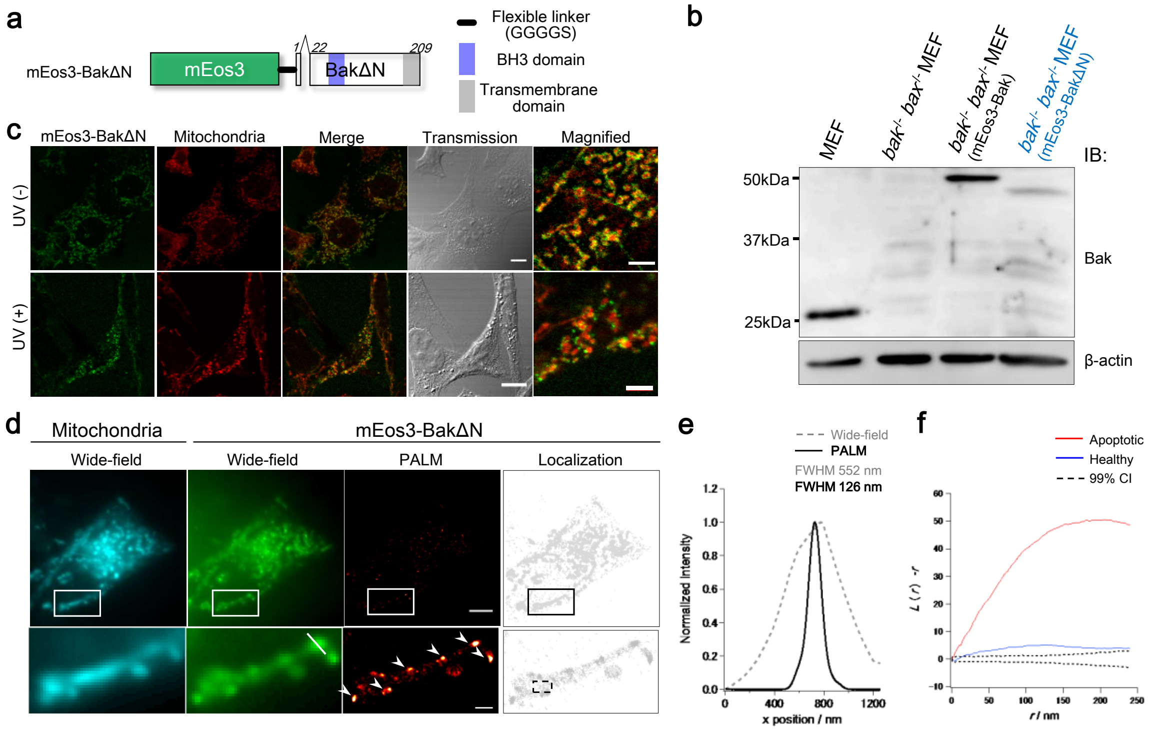

Supplementary Figure 12. Generation of a cell line expressing mEos3-BakΔN for PALM. (a) Schematic construct of mEos3-BakΔN. Numbers in italics show the residue of amino acids in Bak. BakΔN mutant lacks 20 amino acids at the N-terminus. (b) Western blot analysis for investigating the expression of mEos3-BakΔN in the engineered cell line. (c) Fluorescence and transmission images of the cells in the presence or absence of UV irradiation. MitoTracker CMXRos dye was used as a mitochondrial marker. Scale bars: 10 μm (Transmission) or 2 μm (Magnified). (d) PALM imaging of BakΔN clusters in a UV-stimulated cell. Mitochondria were visualized using MitoTracker Deep Red dye under a wide-field configuration. The boxed regions are enlarged (lower panels). Arrows indicate BakΔN clusters. Scale bars: 5 μm (top) or 1 μm (bottom). (e) Line-intensity profile from a line on a BakΔN cluster shown in (d). The cluster size was estimated by calculating FWHM of the profile. (f) Ripley's K analysis for quantitative measurement of the degree of the spatial distribution of mEos3-BakΔN. The analyses were performed in the boxed region (480 nm × 800 nm, black) of an apoptotic cell shown in (d) and a healthy cell (not shown). CI, confidence interval.

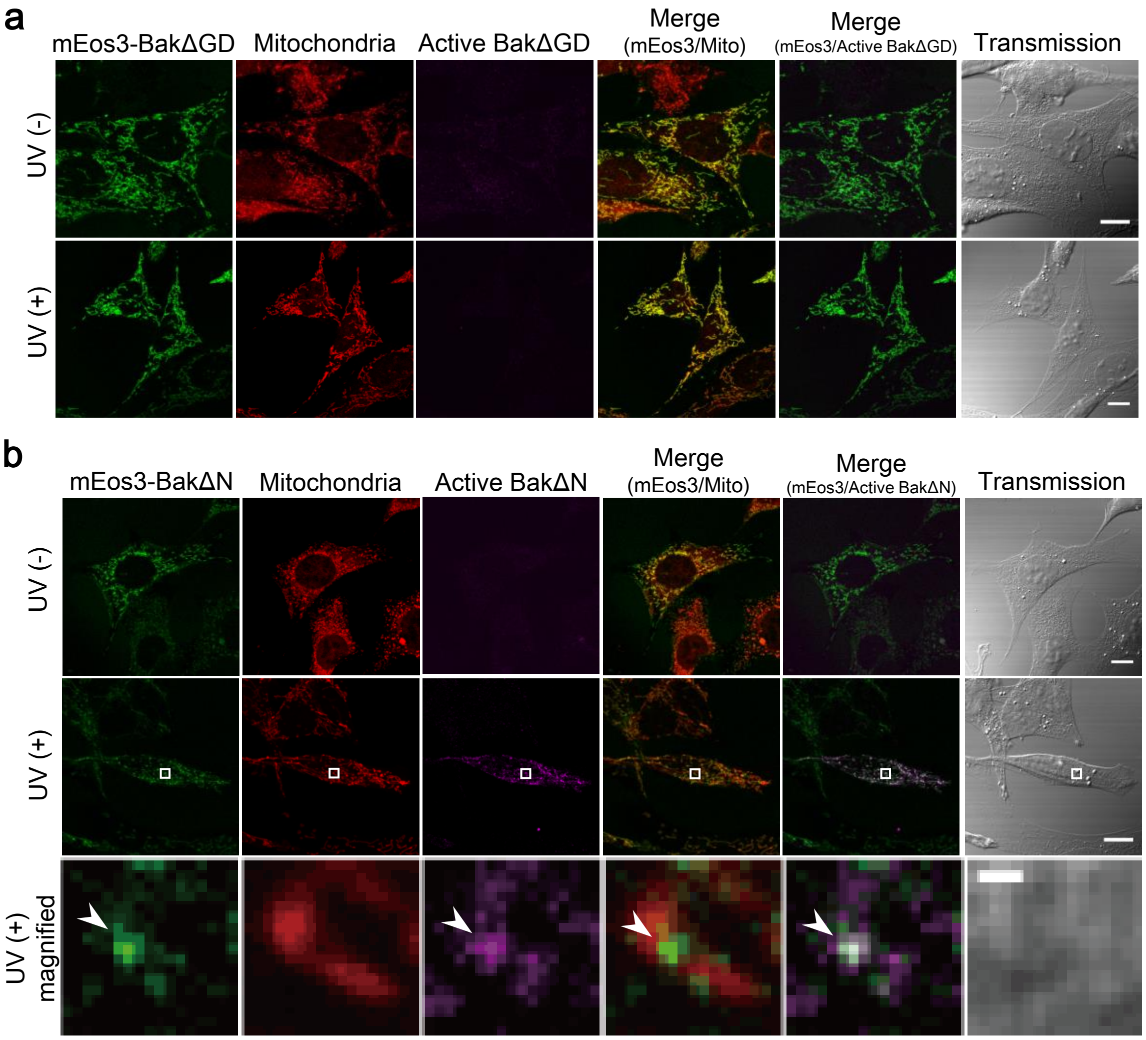

Supplementary Figure 13. Validation of the intramolecular conformational change in mEos3-labeled Bak mutants. (a) Immunostaining of the active BakΔGD connected with mEos3 in the cell. Scale bars: 10 μm. (b) Immunostaining of the active BakΔN connected with mEos3 in the cell. Magnified images in white boxes (middle) are shown at the bottom. Arrowheads indicate a functional BakΔN cluster. Scale bars: 10 μm (top and middle) or 0.5 μm (bottom).

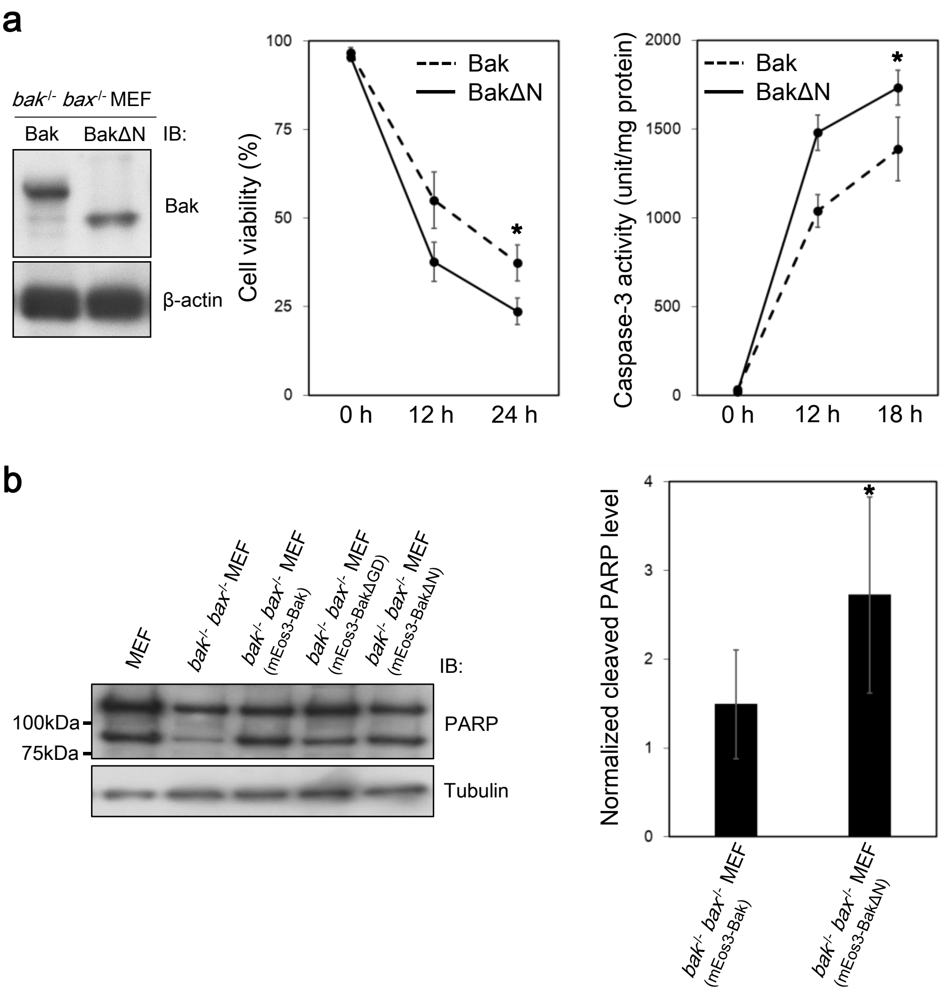

Supplementary Figure 14. Cell death assays. (a) Comparison of viabilities and caspase activities between Bak and BakΔN. Bak or BakΔN was transiently transfected to *bak*$^{-/-}$ *bax*$^{-/-}$ MEF (*left*). Their viabilities and caspase activities after apoptotic stimulation with etoposide were assayed by quantifying Annexin V positive cells and fluorescence intensity of amino-4-methylcoumarin (AMC) released from DEVD-MCA substrate, respectively (*middle* and *right*). One unit was defined as the amount of caspase-3 required to release 1 nmol AMC per min at 37°C. Statistically significant *P* values are denoted by an asterisk, i.e., *$P < 0.05$. Data are represented as mean ± s.d. (n = 3). (b) Western blot analysis for investigating the cleavage of PARP during apoptosis. Cells 24 hours post UV stimulation were subjected to the analysis. Of two bands, upper band represents uncleaved PARP, while lower band indicates cleaved PARP. The graph shows the levels of cleaved PARP which is normalized by internal control and the level of PARP cleavage in *bak*$^{-/-}$ *bax*$^{-/-}$ MEF. Statistically significant *P* value is denoted by an asterisk, i.e., *$P < 0.05$. Data are represented as mean ± s.d. (n = 6).

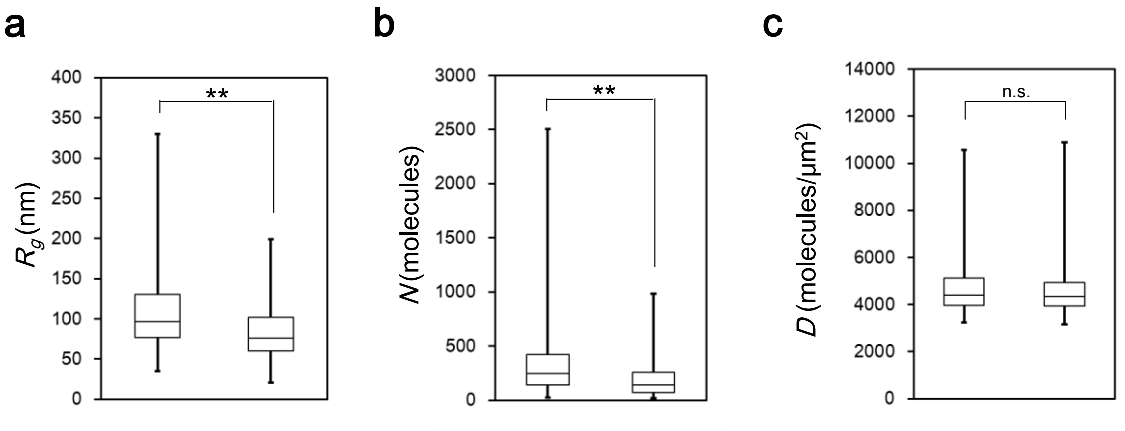

Supplementary Figure 15. Comparison of characteristics between Bak and BakΔN clusters. (a) Box plots of the radius, R_g, of Bak and BakΔN clusters. (b) Box plots of the number of molecules per cluster, *N*, of Bak and BakΔN clusters. (c) Box plots of the molecular density, *D*, of Bak and BakΔN clusters. These plots show the minimum, first quartile, median, third quartile, and maximum. Statistical analyses were performed with the Mann-Whitney test. $P > 0.05$ is indicated as not significant (n.s.). $**P < 0.0001$.

References

36. Ripley B. D., Modelling Spatial Patterns. *J R Stat Soc Ser B* ***39***, 172–212 (1977).

37. Perry, G. L. W. SpPack: spatial point pattern analysis in Excel using Visual Basic for Applications (VBA). *Environ Model Softw* **19**, 559–569 (2004).

38. Baddeley, D., Cannell, M. B. & Soeller, C. Visualization of localization microscopy data. *Microsc Microanal* **16**, 64–72 (2010).

39. Nishida, Y. et al. Discovery of Atg5/Atg7-independent alternative macroautophagy. *Nature* **461**, 654–658 (2009).
